# Unveiling *Candida albicans* intestinal carriage in healthy volunteers: the role of micro- and mycobiota, diet, host genetics and immune response

**DOI:** 10.1080/19490976.2023.2287618

**Published:** 2023-11-28

**Authors:** Margot Delavy, Natacha Sertour, Etienne Patin, Emmanuelle Le Chatelier, Nathaniel Cole, Florian Dubois, Zixuan Xie, Violaine Saint-André, Chaysavanh Manichanh, Alan W. Walker, Lluis Quintana-Murci, Darragh Duffy, Christophe d’Enfert, Marie-Elisabeth Bougnoux, Milieu Intérieur Consortium

**Affiliations:** a Unité Biologie et Pathogénicité Fongiques, Institut Pasteur, Université Paris Cité INRAE, Paris, France; b Human Evolutionary Genetics Unit, Institut Pasteur, Université Paris Cité, CNRS UMR2000, Paris, France; c MGP MetaGénoPolis, INRA, Université Paris-Saclay, Jouy-en-Josas, France; d The Rowett Institute, University of Aberdeen, Aberdeen, UK; e Translational Immunology Unit, Institut Pasteur, Université Paris Cité, Paris, France; f Institut Pasteur, Université Paris Cité, CBUTechS, Paris, France; g Vall d’Hebron Institut de Recerca (VHIR), Vall d’Hebron Hospital Universitari, Vall d’Hebron Barcelona Hospital Campus, Gut Microbiome Group, Barcelona, Spain; h Bioinformatics and Biostatistics HUB, Department of Computational Biology, Institut Pasteur, Université Paris Cité, Paris, France; i APHP, Hôpital Necker-Enfants-Malades, Service de Microbiologie Clinique, Unité de Parasitologie-Mycologie, Paris, France

**Keywords:** *Candida albicans*, colonization resistance, microbiota, mycobiota, GWAS, lifestyle factors, metagenomics, host factors

## Abstract

*Candida albicans* is a commensal yeast present in the gut of most healthy individuals but with highly variable concentrations. However, little is known about the host factors that influence colonization densities. We investigated how microbiota, host lifestyle factors, and genetics could shape *C. albicans* intestinal carriage in 695 healthy individuals from the Milieu Intérieur cohort. *C. albicans* intestinal carriage was detected in 82.9% of the subjects using quantitative PCR. Using linear mixed models and multiway-ANOVA, we explored *C. albicans* intestinal levels with regard to gut microbiota composition and lifestyle factors including diet. By analyzing shotgun metagenomics data and *C. albicans* qPCR data, we showed that *Intestinimonas butyriciproducens* was the only gut microbiota species whose relative abundance was negatively correlated with *C. albicans* concentration. Diet is also linked to *C. albicans* growth, with eating between meals and a low-sodium diet being associated with higher *C. albicans* levels. Furthermore, by Genome-Wide Association Study, we identified 26 single nucleotide polymorphisms suggestively associated with *C. albicans* colonization. In addition, we found that the intestinal levels of *C. albicans* might influence the host immune response, specifically in response to fungal challenge. We analyzed the transcriptional levels of 546 immune genes and the concentration of 13 cytokines after whole blood stimulation with *C. albicans* cells and showed positive associations between the extent of *C. albicans* intestinal levels and *NLRP3* expression, as well as secreted IL-2 and CXCL5 concentrations. Taken together, these findings open the way for potential new interventional strategies to curb *C. albicans* intestinal overgrowth.

## Introduction

*Candida albicans* is an opportunistic pathogen that causes superficial infections, such as vulvovaginal candidiasis, an infection which affects 75% of premenopausal women at least once in their lifetime.^1–3^ In addition, when host defenses are compromised, for example in immunocompromised patients, *C. albicans* can translocate from the gut to the bloodstream and cause systemic infections that are associated with up to 50% mortality.^4–6^

Despite being an opportunistic pathogen, *C. albicans* is primarily a commensal yeast of the gastrointestinal (GI) tract and colonizes up to 95% of the population to various degrees.^7,8^ As an intestinal yeast, *C. albicans* co-exists in the gut with hundreds of other microbial species that comprise the intestinal microbiota,^7,9^ but typically at very low levels.^8^ The microbiota likely plays a role in controlling *C. albicans* growth in the GI tract. In mice, depletion of the bacterial microbiota by antibiotics is necessary to induce colonization by *C. albicans*, whereas non-antibiotic-perturbed mice naturally display colonization resistance to this yeast.^10^ Moreover, in humans, disruption of the bacterial microbiota by antibiotic treatment also results in an overall increase in *C. albicans* levels,^8,11^ thus making antibiotics a risk factor for *C. albicans* intestinal overgrowth. However, we still lack a thorough understanding of other factors that influence *C. albicans* intestinal colonization.

In this study, we investigated how the composition of the bacterial and fungal microbiota and the host’s diet, medical history, and environment correlated with *C. albicans* carriage in healthy adults using data collected from 695 healthy volunteers from the *Milieu Intérieur* cohort.^12^ We also conducted a genome-wide association study (GWAS) to identify genetic factors associated with host susceptibility to *C. albicans* colonization. Finally, we investigated the interplay between the extent of *C. albicans* intestinal carriage and host immune response upon *ex vivo* stimulation with *C. albicans* heat-killed cells.

## Results

### Characterization of the mycobiota of a large cohort of healthy subjects

**Figure 1.**
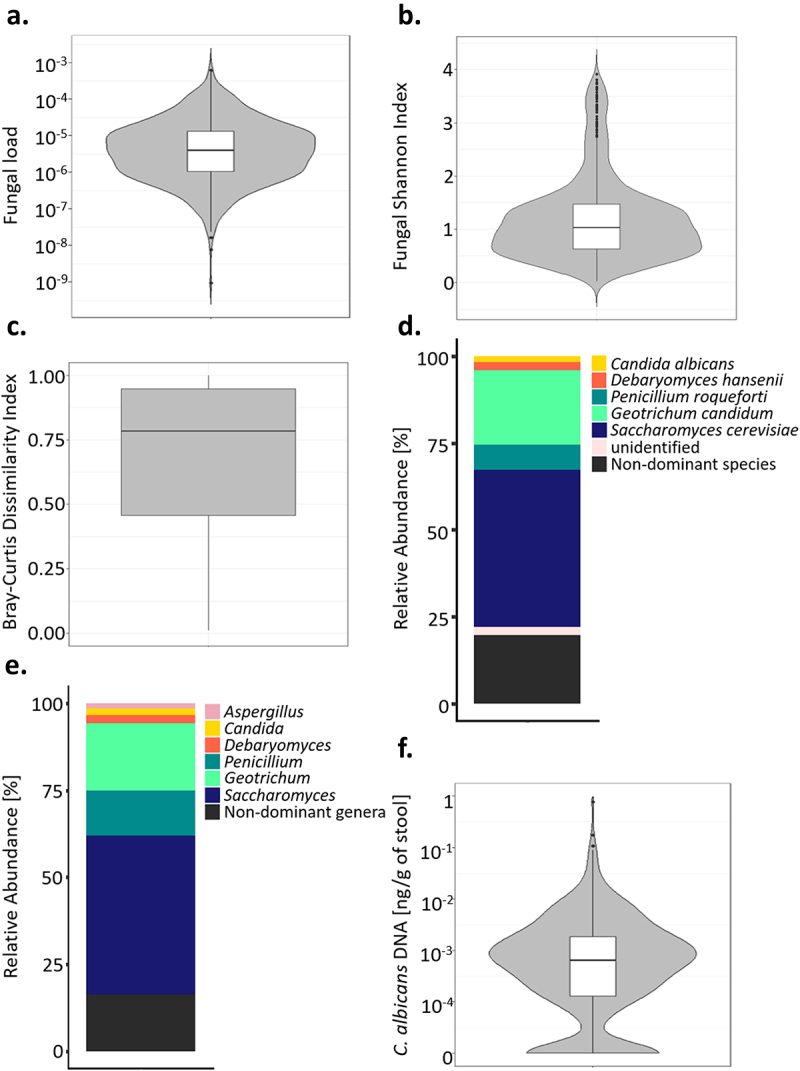
Mycobiota characteristic of healthy subjects. (a). Violin plot of the fungal load observed in the 695 healthy subject fecal samples. (b). Alpha diversity : violin plot of the Shannon Index obtained for 604 healthy subjects’ fecal samples. (c). Beta diversity: Bray-Curtis dissimilarity values between samples donated by different subjects for ITS2 sequencing data obtained for 604 healthy subjects. Values range from 0 to 1, with 0 being the least dissimilar and 1 being the most dissimilar. (d). Barplot of the mean relative abundances of the fungal species that were detected in at least 50% of the 604 studied healthy subjects with a relative abundance above 0.1%. (e). Barplot of the mean relative abundances of the fungal genera that were detected in at least 50% of the 604 studied healthy subjects with a relative abundance above 0.1%. (f) Violin plot of *C.* *albicans* DNA levels observed in the 695 healthy subject fecal samples.

To describe the main characteristics of healthy intestinal mycobiota, we analyzed a fecal sample from each of the 695 healthy adults from the *Milieu Intérieur* cohort. First, we quantified the fungal load, which is the ratio of the fungal DNA concentration to the total fecal DNA concentration of a given sample (see Materials and Methods). Fungi represented only a small fraction of the total intestinal microbiota with a median fungal load of 4.0 × 10^−6^ (min: 9.0 × 10^−10^, max: 6.26 × 10^−4^, Figure 1a).

Using ITS2-targeted sequencing, we further characterized the mycobiota of 604 of the *Milieu Intérieur* healthy subjects. The mycobiota of the subjects was characterized by a rather low richness and evenness, compared to what is usually observed for the bacterial microbiota,^7,13^ with a median Shannon Index of 1.03 (min: 0.02, max: 3.91, Figure 1b) and a median of 35 different amplicon sequence variants (ASVs) per sample. Furthermore, the mycobiota was highly dissimilar between the subjects, with a median β-diversity, estimated by Bray-Curtis dissimilarity index, of 0.78 (min:0.01, max:1.00, Figure 1c).

ITS2 sequence analysis resulted in 2488 fungal amplicon sequence variants (ASVs). 2461/2488 (98.9%) were annotated at the phylum level, 1521 (61.1%) at the genus level, and 539 (22.6%) at the species level. Overall, the 1521 ASVs annotated at the genus level and the 539 ASVs annotated at the species level represented 96.9% and 95.7% of the total number of sequences, respectively. Most of the fungal ASVs belonged to the Ascomycota (mean relative abundance of 97.3%), the Basidiomycota (1.9%), and the Mucormycota (0.8%, Supplementary Fig. S1). Among the 317 fungal species annotated, five were present in more than 50% of the subjects, with a relative abundance above 0.1%: *Saccharomyces cerevisiae, Geotrichum candidum, Penicillium roqueforti, Debaryomyces hansenii* and *C. albicans* (Figure 1d, Supplementary Table S1). Three hundred and fifty fungal genera were annotated, among which *Saccharomyces, Geotrichum, Penicillium, Debaryomyces, Candida* and *Aspergillus* were the dominant fungal genera, being detected in at least 50% of the subjects with a relative abundance above 0.1% (Figure 1e). The remaining 344 fungal genera are referred to in this study as “non-dominant genera.”

**Figure 2.**
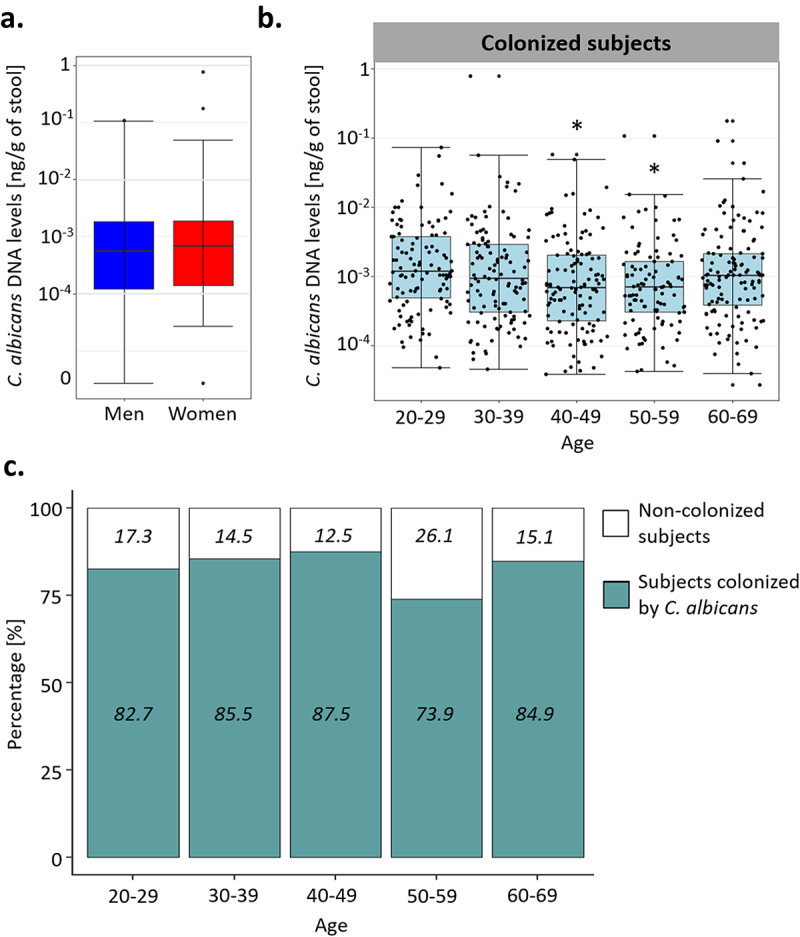
Effect of the age and sex of the subjects on *C. albicans* carriage. (a). Boxplot of the distribution of *C. albicans* DNA levels in male and female subjects. (b). Boxplot of the distribution of *C. albicans* DNA levels depending on the age of the subjects in the subjects colonized by *C. albicans*. (c) Percentage of subjects colonized by *C. albicans* (green) and not colonized (white) within each age group. The percentage of colonized and not colonized subjects within each age group are noted in italics *p-value < .05.

Using qPCR, we quantified the absolute abundance of *C. albicans* in the fecal samples of these volunteers. We detected *C. albicans* DNA in 576/695 subjects (82.9%), in concentrations ranging from 2.7 × 10^−5^ to 0.78 ng/g of stool, with a median of 8.95 × 10^−4^ ng/g (Figure 1f). *C. albicans* DNA concentrations and colonization rates were similar between the sexes (Wilcoxon test; p-value of 0.44, Chi-square test; p-value of 0.59, Figure 2a), but we observed dissimilarities based on the age of the volunteers, both for the colonization rates and *C. albicans* levels (One-way ANOVA; q-value of 0.008, chi-square test; p-value of 0.025, Figure 2b,c). Only 73.9% of the subjects between 50 and 59 were colonized with *C. albicans*, whereas 85.2% of the rest of the cohort was colonized by this yeast. We also observed differences in *C. albicans* carriage between the age groups of subjects who were colonized by *C. albicans*. Indeed, subjects between 20 and 29 years old carried, on average, higher intestinal *C. albicans* levels than subjects from the to 40–49 (Tukey HSD; q-value of 0.025) and 50–59 (Tukey HSD; q-value of 0.039) age groups (Figure 2b).

### *Identification of microbial species with potential anti-*C. albicans *activity*

Considering the wide variations in *C. albicans* carriage observed in the *Milieu Intérieur* healthy subjects, we hypothesized that the composition of bacterial and fungal microbiota could at least partly explain these variations. Therefore, we aimed to identify bacterial and fungal species that were negatively or positively associated with *C. albicans* carriage and could thus either inhibit or, to the contrary, promote *C. albicans* growth in the human gut.

In the first step, by taking advantage of the previously generated whole-genome shotgun metagenomic sequence data available from the Milieu Intérieur cohort,^13^ we used MaAsLin2 (Microbiome Multivariable Associations with Linear Models^14^ to search for associations between *C. albicans* levels, deduced from qPCR quantification, and bacterial species abundance, deduced from the shotgun metagenomics data and annotated at the species level. Of the 231 metagenomic species analyzed, *Intestinimonas butyriciproducens* was the only species that was significantly associated with *C. albicans*, highlighting its possible anti-*C. albicans* activity (MaAsLin2; q-value of 0.029, association coefficient of −0.11).

Since *Intestinimonas butyriciproducens* produces butyrate,^15^ a molecule known to inhibit *C. albicans* growth and hyphae production,^16,17^ we tested the effect of culture supernatants from this species on *C. albicans* growth and morphogenesis. We also tested the effect of the supernatants of several additional bacterial species that we highlighted as potentially interesting in preliminary studies: *Bacteroides massiliensis, Bacteroides ndongoniae, Coprobacter secundus, Enorma massiliensis, Pseudoflavonifractor capillosus, Lactococcus lactis* and *Roseburia intestinalis*. Since *L. lactis* is a facultative anaerobe, *L. lactis* supernatant was tested after it was grown under anaerobic and aerobic conditions. We used *Bifidobacterium adolescentis*, strain L2–32 as a *C. albicans* inhibition control, since this strain has been recently shown to have a strong inhibitory effect on both *C. albicans* growth and morphology.^18^

**Figure 3.**
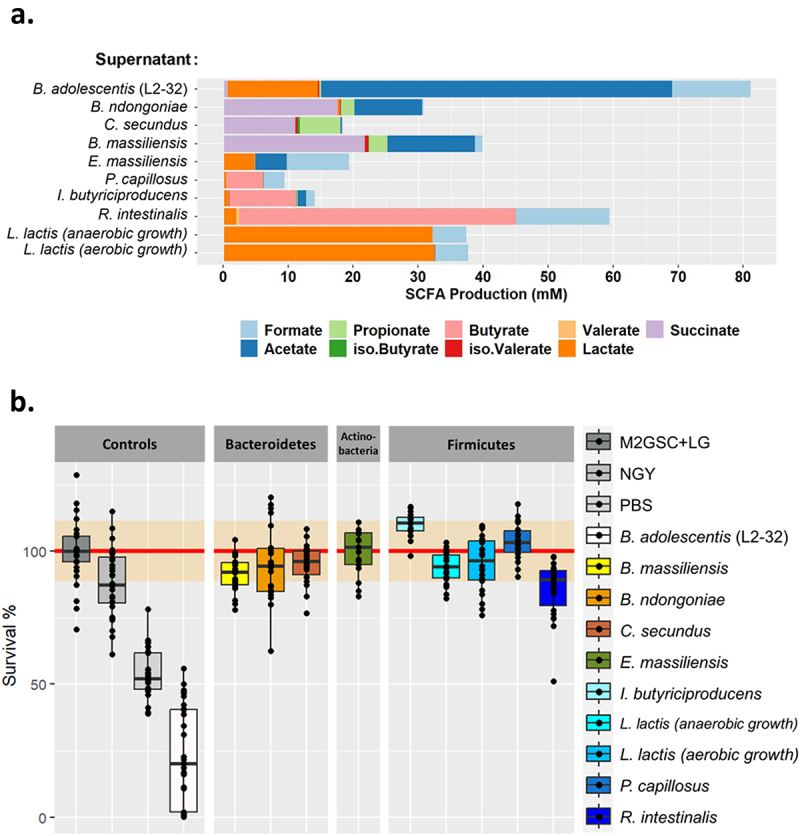
Characterization of bacterial species supernatants and their effect on *C. albicans* growth. (a). Characterization of the short-chain fatty acids (SCFA) content of each of the bacterial species tested. The SCFA were quantified in the culture supernatants and normalized against the background growth medium control. (b). Boxplot representing the effect of bacterial culture supernatants on the survival of *C. albicans*, strain SC5314 after 24 h of exposure, relative to the control growth in M2GSC medium (red line, confidence intervals are represented in orange). NGY: *C. albicans* growth in NGY medium, PBS: *C. albicans* growth in PBS.

All of these bacteria are short-chain fatty acid (SCFA) producers, but the concentration and type of SCFA varied greatly between the species (Figure 3a). In agreement with a previous study,^18^ *B. adolescentis* was the largest producer of SCFA, especially acetate, whereas *R. intestinalis* was the species that produced the largest amount of butyrate, with an average of 42.5 mM (Figure 3a). However, except for *B. adolescentis*, none of the supernatants of the tested species were able to inhibit *C. albicans* growth *in vitro* (Figure 3b). Similarly, none of the studied bacterial species’ supernatants had any effect on *C. albicans* morphology, as observed by microscopy, except for *R. intestinalis*, for which we observed a slight reduction in *C. albicans* hyphae formation after exposure to *R. intestinalis* supernatant (Supplementary Fig. S2).

**Figure 4.**
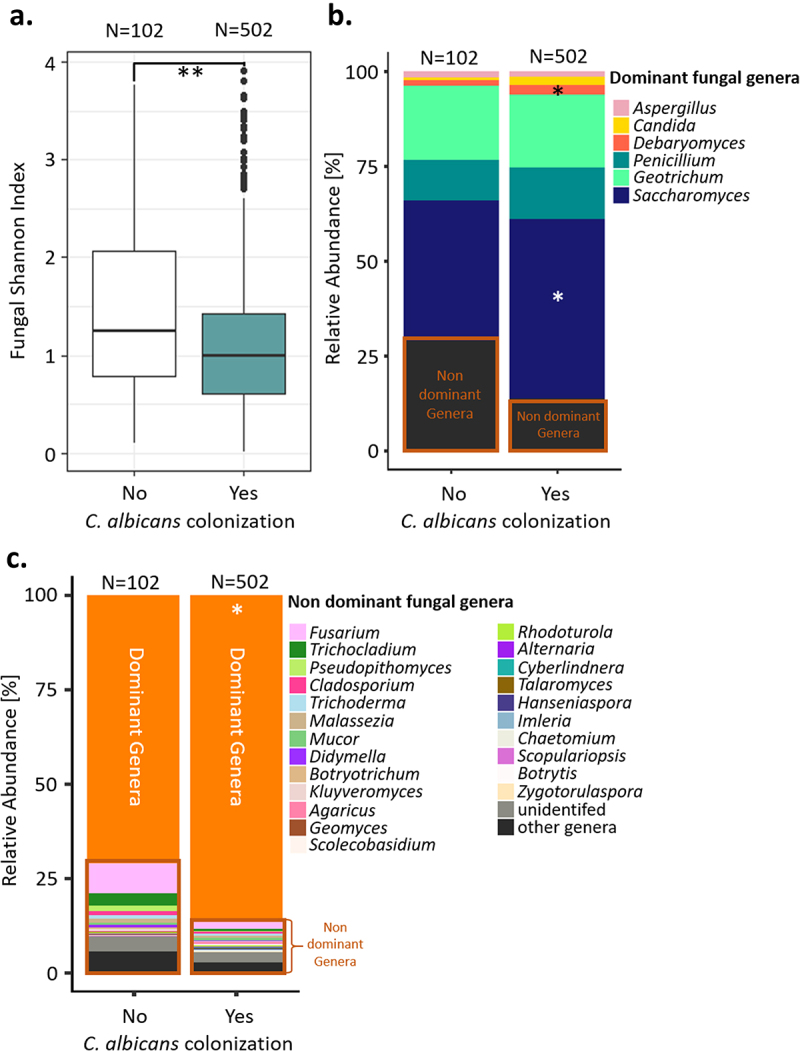
Comparison of the gut mycobiota of the Milieu intérieur subjects colonized by *C. albicans* with that of non-colonized Milieu Intérieur subjects. (a). Boxplot of the distribution of fungal Shannon index depending on *C. albicans* colonization state. (b). Barplot of the mean relative abundances of the fungal species that were detected in at least 50% of the studied healthy subjects with a relative abundance above 0.1%, depending on *C. albicans* colonization state. (c) Barplot of the mean relative abundances of the non-dominant fungal genera whose mean relative abundance, across subject, was above 0.1%, depending on *C. albicans* colonization state. Dominant genera are fungal genera detected in at least 50% of the studied healthy subjects with a relative abundance above 0.1%. Non-dominant genera are the remaining fungal genera. *p-value < .05, **p-value < .005.

While the extent of *C. albicans* intestinal carriage may be associated with the composition of the bacterial componentof the gut microbiota, competition by the mycobiota may also be important. Indeed, the *Milieu Intérieur* subjects in whom *C. albicans* DNA could be detected by qPCR were characterized by a lower fungal α-diversity, as estimated by the Shannon Index, than the subjects that were not colonized (Wilcoxon test; p-value of .00069, Figure 4a). We also observed differences in the relative abundances of the dominant fungal genera in the subjects depending on whether they were colonized by *C. albicans*, with the relative abundances of *Debaryomyces* spp. and *Saccharomyces* spp. being reduced in the non-colonized subjects (Wilcoxon test; q-values of 0.008, 0.043, and 0.010, respectively; Figure 4b). The overall relative abundance of the non-dominant fungal genera was also increased in subjects that were not colonized by *C. albicans* (Wilcoxon test, p-value of .00037, Figure 4b,c).

In order to identify specific fungal species associated with *C. albicans* intestinal carriage, we used MaAsLin2^14^ to compare the relative abundances of the different fungal species, as deduced from the ITS2 sequencing data, in the *Milieu Intérieur* subjects that were colonized by *C. albicans* to those that were not. Four fungal species were significantly associated with *C. albicans*, all negatively: *Penicillium oxalicum*, *Fusarium falciforme*, *Trichocladium gilmaniellae* and *Trichocladium seminis citrulli* (Table 1).

**Table 1.** Fungal species associated with *C. albicans* colonization in the Milieu Intérieur subjects. The prevalence of each fungal species within the subjects non-colonized by *C. albicans* and within the subjects colonized by *C. albicans* is represented in percentage and in ratio.

	Prevalence [%] of each of the four fungal species in Milieu Intérieur		MaAsLin2	
	Non colonized by *C. albicans*	Colonized by *C. albicans*	P value	FDR
*Penicillium oxalicum*	27.4 28/102	6.4 32/502	.00032	0.021
*Fusarium falciforme*	31.4 32/102	10.0 50/502	.0020	0.035
*Trichocladium gilmaniellae*	29.4 30/102	8.2 41/502	.0021	0.035
*Trichocladium seminis citrulli*	27.4 28/102	7.2 36/502	.0018	0.035

### *Diet, medical and environmental factors have a limited impact on* C. albicans *growth*

As the gut microbiota composition seemed to have a limited association with *C. albicans* colonization, we investigated whether a specific dietary pattern, medical history, or a combination of environmental factors might be associated with *C. albicans* carriage in the human gut. To test this hypothesis, we combined linear mixed models and multiway ANOVA, adjusted for age, sex, and technical variables (see Materials and Methods). In total, we analyzed 201 variables, including 12 demographic variables (i.e., physical activity, housing conditions, city of origin, etc.), 46 diet-related variables (i.e., consumption frequency of major food groups, number of meals per day, etc.), six basic physiological measures (i.e., weight, body mass index (BMI), blood pressure, etc.), 70 variables related to the subject or subject’s family medical history, 44 laboratory measures (i.e., creatinine, gamma GT, serology, etc.), and 30 variables related to the subjects’ sleeping, drugs and smoking habits, and socio-professional information. The list of 201 variables analyzed is available in the Supplementary Data (Supplementary Table S2).

**Figure 5.**
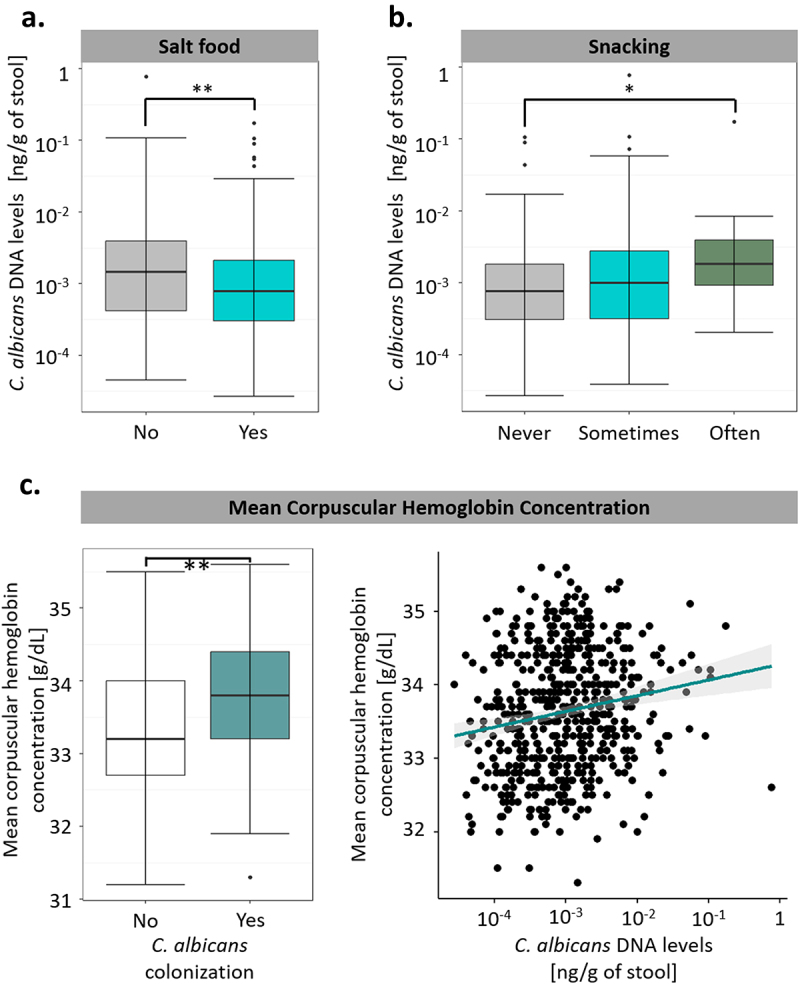
Diet and medical factors have a limited impact on *C. albicans* intestinal carriage and colonization. (a). Boxplot of the variation of *C. albicans* DNA levels according to the salting habits of the subjects. (b). Boxplot of the variation of *C. albicans* DNA levels according to the snacking habits of the subjects. (c). Association between *C. albicans* intestinal carriage and colonization and the mean corpuscular hemoglobin concentration. (left) Boxplot of the distribution of the mean corpuscular hemoglobin concentration depending on *C. albicans* colonization state. (right) scatterplot of the mean corpuscular hemoglobin concentration relative to intestinal *C. albicans* DNA levels. The regression line is represented in green, and the interval of confidence in gray. *p-value < .05, **p-value < .005.

Certain dietary factors were correlated with *C. albicans* growth, with the subjects’ salt consumption being negatively associated with *C. albicans* intestinal levels (q-value of 0.0047, Figure 5a), whereas the subjects’ snacking habits (q-value of 0.016, Figure 5b) were associated with a higher *C. albicans* intestinal concentration. Surprisingly, the only biological variable we identified in this analysis was the mean corpuscular hemoglobin concentration of the subjects, which was strongly positively associated with both *C. albicans* DNA levels and *C. albicans* colonization (q-value of 0.00064 and 0.0010, respectively, Figure 5c). However, we did not observe any significant correlations between environmental variables, such as housing condition or exposure to secondhand smoke, and *C. albicans* intestinal levels or colonization. Moreover, with the exception of the mean corpuscular hemoglobin concentration, we did not identify any significant associations with *C. albicans* colonization, suggesting that although diet and lifestyle might contribute to the extent of *C. albicans* carriage in colonized subjects, these factors do not play any role in host susceptibility or resistance to *C. albicans* colonization.

### *Genome-wide association study identifies a suggestive* C. albicans *gastrointestinal colonization susceptibility locus on chromosome 20*

**Figure 6.**
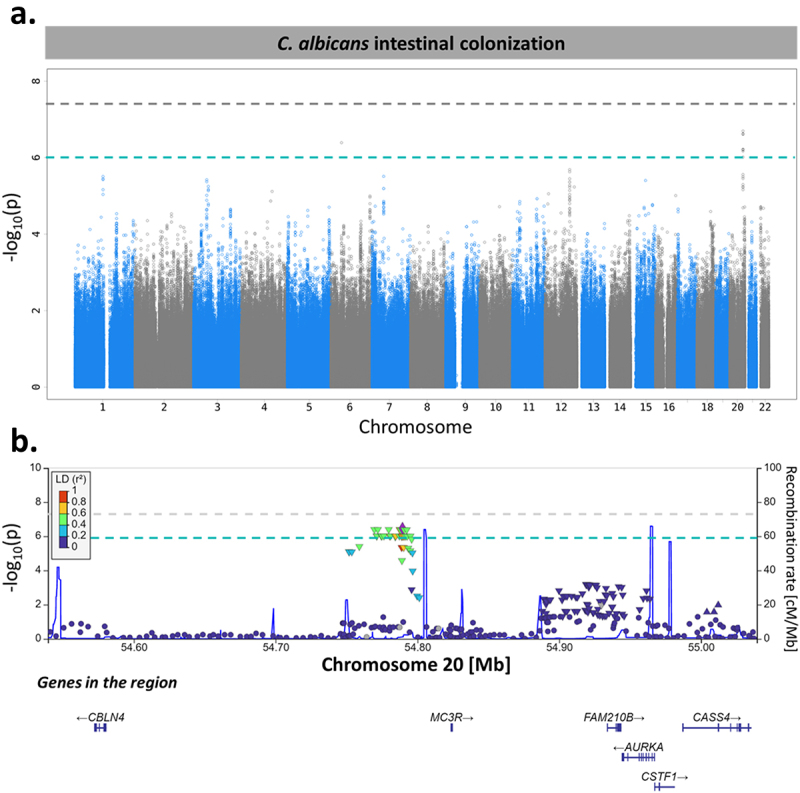
*MC3R* locus is associated with *C. albicans* intestinal colonization susceptibility. (a). Manhattan plot of single-nucleotide polymorphisms (SNPs) associated with *C. albicans* intestinal colonization susceptibility, identified by the genome-wide association study (GWAS) conducted on the 695 subjects of Milieu Intérieur. The x-axis represents the chromosomal position, and the y-axis represents the -log_10_(p-values) associated with each SNP. The green line represents the suggestive threshold for association (p-value <1.00 × 10^−6^). The gray line represents a threshold of 5.00 × 10^−8^. (b). Regional association plot for the *C. albicans* intestinal colonization-associated SNP, rs2870723 (purple diamond). Each dot represents a SNP, the color of the dots corresponds to the linkage disequilibrium of the neighboring SNPs with the top SNP. The x-axis represents the chromosomal position, the left y-axis represents the -log_10_(p-values) associated with each SNP (dots) and the right y-axis represents the recombination rate (blue line) occurring in each position of the locus.

To investigate the effects of genetic variants on *C. albicans* gut colonization susceptibility, we compared the genotype profiles^19^ of 576 *Milieu Intérieur* subjects who were colonized by *C. albicans* to those of 119 *Milieu Intérieur* subjects who were not. After quality control^19^ and genotype imputation, we obtained a total of 5,677,102 single-nucleotide polymorphisms (SNPs) that were tested for association with *C. albicans* intestinal colonization using linear mixed models. The models were adjusted for age, sex, smoking habits, and genetic relatedness among subjects, as described by Patin *et al*. 2018.^19^ From this, we identified 26 SNPs in two independent loci that showed a suggestive association with *C. albicans* colonization (p-value <1.00 × 10^−6^, Figure 6a).

**Figure 7.**
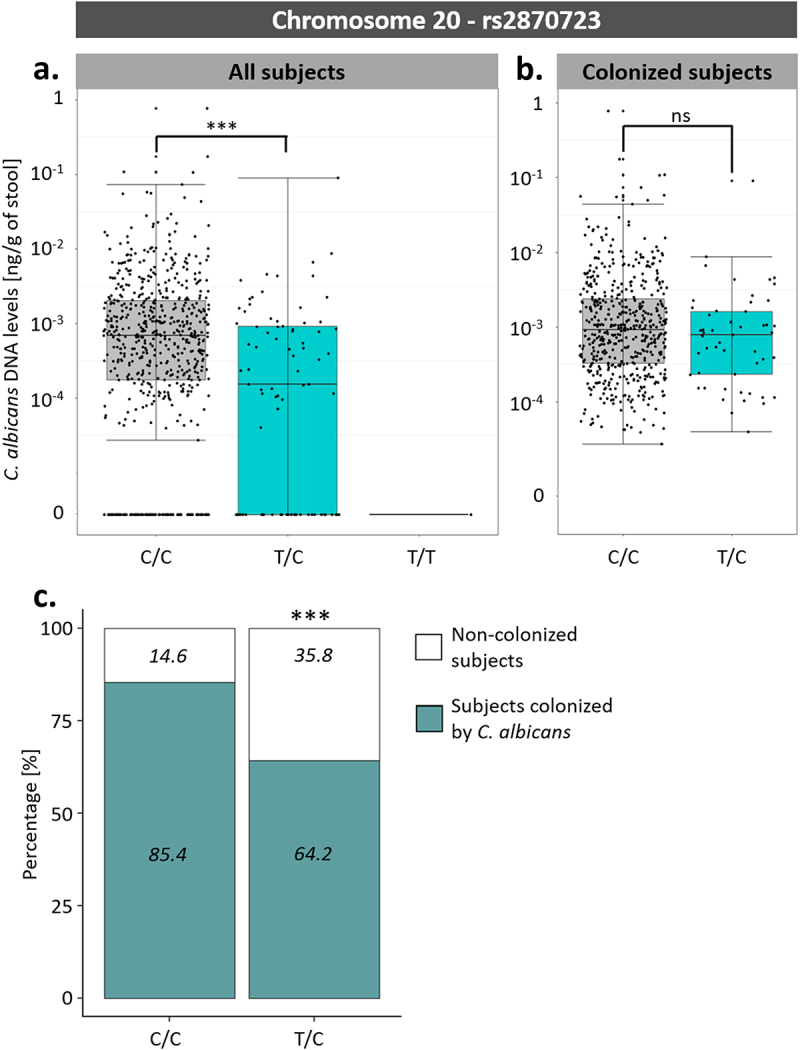
Association between rs2870723 genotypes and the levels of *C. albicans* intestinal carriage. (a) Boxplot of the variation of *C. albicans* DNA levels according to the rs2870723 genotype of the 695 Milieu Intérieur subjects. (b) Boxplot of the variation of *C. albicans* DNA levels according to the rs2870723 genotype of the 574 subjects colonized with *C. albicans*. (c) Percentage of subjects colonized by *C. albicans* (green) and not colonized (white) according to the rs2870723 genotype of the Milieu Intérieur subjects. The percentage of colonized and not colonized subjects within each genotype are noted in italics. ***p-value < .0005, ns non-significant.

Among these associations, an SNP on chromosome 20, rs2870723, showed the strongest association with susceptibility to *C. albicans* intestinal colonization (p-value of 3.43 × 10^−7^, β coefficient of −0.224). Although rs2870723 genotypes were associated with *C. albicans* intestinal levels when considering all 695 subjects (Figure 7a; One-way ANOVA; p-value of 2.72 × 10^−7^), this was not the case when considering only the 576 subjects colonized by *C. albicans* (Figure 7b; One-way ANOVA; p-value of .10). Therefore, rs2870723 is associated with host susceptibility to *C. albicans* colonization (Chi-square test; p-value of 1.06 × 10^−6^, Figure 7c) but not with the extent of *C. albicans* carriage in colonized subjects.

Interestingly, Rs2870723 is located relatively close to the Aurora Kinase A (*AURKA*) gene (Figure 6b), in which several SNPs have been associated with the mean corpuscular hemoglobin concentration, a variable that we identified as being significantly associated with *C. albicans* colonization. Rs2870723 is also located close to *MC3R* (Figure 6b), a gene that encodes a melanocortin receptor, whose mutations have been previously associated with obesity.^20–23^ However, we did not identify any association between subject weight and *C. albicans* intestinal colonization in the Milieu Intérieur subjects.

### *Higher* C. albicans *intestinal levels are associated with the immune response after* C. albicans *blood stimulation*

As seen in the previous part of this study, the factors underlying *C. albicans* colonization in healthy individuals are multifactorial, and several medical, diet-related, or genetic factors might contribute to shaping the extent of *C. albicans* carriage. Since more than 80% of the healthy population is colonized by *C. albicans*, even though this yeast can become highly pathogenic in immunocompromised patients, we wondered if there was an advantage to the host in maintaining higher levels of *C. albicans* in the gut. In particular, we hypothesized that a higher carriage of *C. albicans* could contribute to host protection in cases of systemic infection.

To test this hypothesis, we searched for associations between the extent of *C. albicans* carriage in subjects colonized by this yeast, the level of expression of 546 immune genes, and the production of 13 cytokines before and after whole blood stimulation with heat-killed *C. albicans* cells. We used linear mixed models adjusted for age, sex, immune cell proportions, and technical variables. In addition, the models were adjusted for the genotype of the rs12567990 SNP, as we have previously shown that this SNP, located at the *CR1* locus, regulates immune gene expression after *C. albicans* blood stimulation.^24^

**Figure 8.**
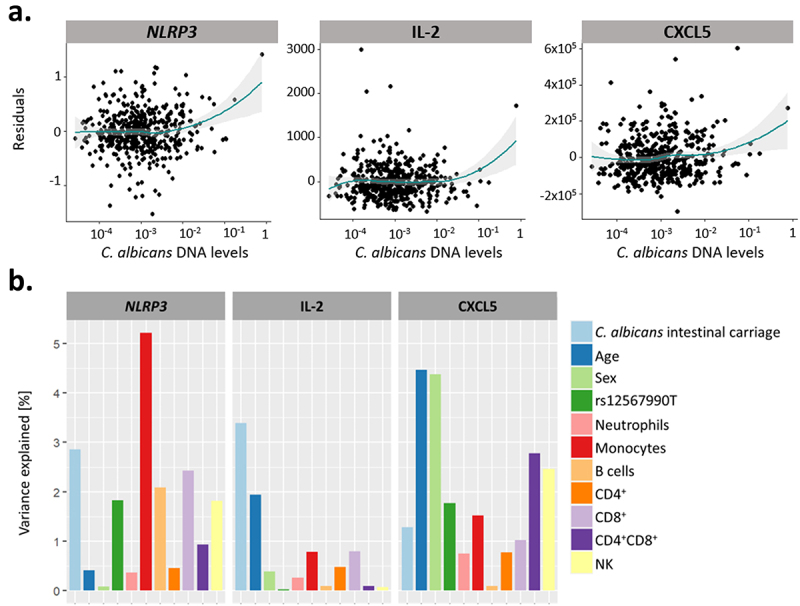
The extent of *C. albicans* intestinal carriage was associated with the expression levels of *NLRP3* and the concentration of IL-2 and CXCL5 upon ex vivo *C.* *albicans* blood stimulation. (a). Residual plots of the association between *C. albicans* intestinal DNA levels and the expression of *NLRP3*, and the concentration of IL-2 and CXCL5 upon *C. albicans* stimulation. Linear model residuals are plotted in relation to the expression of *NLRP3* or the concentration of IL-2 and CXCL5. The LOESS line is represented in green and the interval of confidence in gray. (b) Proportion of the expression and concentration variance explained by *C. albicans* intestinal carriage, age, sex, genetics, and proportions of immune cells for *NLRP3*, IL-2 and CXCL5, in response to *C. albicans* blood stimulation.

None of the 546 genes and 13 cytokines were associated with the extent of *C. albicans* carriage under non-stimulated conditions, suggesting that *C. albicans* intestinal carriage is independent of host immunity at baseline. However, we identified positive associations between the extent of *C. albicans* intestinal carriage and the expression level of *NLRP3* (q-value of 0.036) and the concentrations of IL-2 (q-value of 0.00059) and CXCL5 (q-value of 0.029) upon blood stimulation with *C. albicans* heat-killed cells (Figure 8a). Variability in the *NLRP3* response after *C. albicans* stimulation was mainly determined by the proportion of monocytes, with *C. albicans* intestinal levels being the second main contributing factor, explaining 2.86% of the variance (Figure 8b). Variability in the CXCL5 response was influenced by the age and sex of the subjects (1.28% of the variance explained by the extent of *C. albicans* intestinal carriage, Fig. Figure 8b). However, IL-2 production upon *C. albicans* blood stimulation was influenced by the extent of *C. albicans* carriage, which was associated with the highest percentage of variance (3.39%; Figure 8b). It should be noted that these associations were specific to blood stimulation by *C. albicans*. Indeed, *C. albicans* intestinal levels were not associated with *NLRP3* expression, IL-2, and CXCL5 levels after blood stimulation with *Escherichia coli* (*NLRP3*: q-value of 0.17, IL-2: q-value of 0.60, CXCL5: q-value of 0.46), *Staphylococcus aureus* (*NLRP3*: q-value of 0.61), *Bacillus* Calmette-Guérin (BCG, q-value of 0.45, 0.24, and 0.24), influenza A live virus (q-value of 0.58, 0.99, and 0.99), or the staphylococcal enterotoxin B superantigen (q-values of 0.35, 0.87, and 0.28).

## Discussion

In this study, we explored different factors that could potentially modulate the presence or intensity of the intestinal carriage of *C. albicans* in healthy subjects. First, we showed that *C. albicans* was present, with large inter-subject variability, in the gut of 82.9% of the healthy subjects studied. This variability could not be explained by the subjects’ sex, since men and women carried similar intestinal levels of *C. albicans*. Interestingly, subjects aged between 50 and 59 years were less colonized than other individuals, suggesting a non-linear effect of age on *C. albicans* intestinal colonization. However, considering that all subjects were recruited during the same period, this decrease in *C. albicans* colonization might be associated with this specific generation of subjects rather than being related to the specific age of the subject at the time. This study is the first to assess *C. albicans* carriage using qPCR in a large population. A recent study published by our group has already reported a high carriage of *C. albicans* in healthy adults,^8^ thus confirming that *C. albicans* might not be a facultative commensal, as previously thought, but that it is able to maintain itself in the GI tract of most individuals, often at very low levels.

Nonetheless, the prevalence of *C. albicans* intestinal colonization may have a genetic component. Indeed, we identified 26 SNPs located at two independent loci that are associated with *C. albicans* colonization. In particular, rs2870723, an SNP located on chromosome 20, was strongly associated with host susceptibility to *C. albicans* colonization, but not with the levels of *C. albicans* carriage in subjects colonized by this yeast. This SNP is located between *RNA5SP487*, an RNA 5S pseudogene that has not been widely described, and *MC3R*, which encodes a melanocortin receptor. Considering the phenomenon of Gene Linkage Disequilibrium, referring to the fact that alleles from closely located genes are more likely to be transmitted together than alleles from distant genes, it might be interesting to further explore neighboring genes.

Considering that mutations in *MC3R* have been associated with obesity in genetics^20–22^ and *in vivo* ^23^ studies, it is possible that this genetic association with *C. albicans* colonization results from an indirect interaction with subject weight. Especially since *C. albicans* has been reported to be more abundant in overweight people.^25^ However, we did not identify any association between subject weights and *C. albicans* intestinal colonization in the *Milieu Intérieur* subjects, which might be explained by the fact that all subjects were healthy volunteers withno extreme BMI. Polymorphisms in *MC3R* have also been associated with increased susceptibility to tuberculosis, probably through mediation of the inflammatory response.^26–28^ Thus, it is possible that the identified SNPs resulted in a differentiated inflammatory and/or inhibitory response against *C. albicans*.

Moreover, an SNP in the *AUKRA* gene, another neighboring gene of rs2870723, has been associated with mean corpuscular hemoglobin concentration in previous GWAS studies,^29,30^ a variable that was found to be associated with *C. albicans* intestinal carriage and colonization susceptibility. Therefore, the association between rs2870723 and *C. albicans* intestinal colonization might have originated from an interaction between this SNP and *AURKA* gene.

Notably, none of the identified SNPs reached the typically used genome-wide significant p-value threshold (p-value <5 × 10^−8^). However, we need to consider the fact that 695 subjects are a rather low number for a GWAS study, especially considering that only 119 of those subjects were not colonized by *C. albicans*. Therefore, we probably lacked statistical power. Thus, this study should be replicated in larger independent cohorts to confirm and better decipher the potential role of rs2870723 in host susceptibility to *C. albicans* colonization.

We also found evidence to suggest that the host microbiota and mycobiota composition might contribute to *C. albicans* carriage, identifying the bacterium *Intestinimonas butyriciproducens*, a butyrate-producer, and four fungal species, *P. oxalicum, F. falciforme, T. gilmaniellae* and *T. seminis citrulli*, as potential anti-*C. albicans* signatures. In addition, we found that the relative abundance of two dominant fungal genera, *Saccharomyces* and *Debaryomyces*, was increased in subjects colonized by *C. albicans*. The increase in *Saccharomyces* spp. abundance is particularly interesting because, within *Milieu Intérieur* donors, this genus is mainly composed of *S. cerevisiae*. The abundance of this fungal species has been previously shown to decrease in patients suffering from inflammatory bowel disease (IBD), while *C. albicans* abundance tends to increase, thus suggesting a negative association between the two fungal species.^31,32^ This discrepancy between these results and our findings suggests that although competition between *C. albicans* and *S. cerevisiae* may arise in the context of a disease, such competition is not relevant in the healthy intestinal niche.

Despite being negatively associated with *C. albicans* carriage *in silico*, we were unable to show an effect of culture supernatants from *I. butyriciproducens* on the growth or morphology of *C. albicans*. It is possible that the *in vitro* experimental conditions used were not optimal for the growth of this bacterium and/or subsequent release of SCFAs, such as butyrate, or other antifungal metabolites. Indeed, our analyses showed a relatively low concentration of SCFAs in the supernatant of this bacterium (Figure 3a), which could increase if the bacterium was grown under alternative growth conditions. Moreover, the release of antifungal metabolites, such as SCFA, is not the only method by which microbial species can inhibit fungal growth. Indeed, a bacterial species can also inhibit *C. albicans* growth by modulating the host immune response^10,16,33^ or by competing for niches, adhesion sites, and/or nutrients.^34–36^ Finally, *C. albicans* inhibition by *I. butyriciproducens*, might be strain-specific, as it has been reported for *B. adolescentis*.^18^ Alternatively, the lack of activity observed *in vitro* might indicate that *I. butyriciproducens* has no activity against *C. albicans* and that the metagenomics-based anti-correlation occurred by chance, or even that the correlation is the consequence of an effect of *C. albicans* against this bacterium. Nevertheless, this study demonstrates the importance of testing *in silico*-based predictions in the laboratory, as a dual approach offers the best prospect for identifying novel anti-*Candida* therapeutics (see Delavy *et al*. 2023).^37^

Regarding other potential factors that might influence *C. albicans* colonization levels, the subjects in this study that reported having a low-sodium diet and those that reported frequent snacking between meals carried, on average, higher intestinal levels of *C. albicans*. This is not surprising since diet is known to strongly modulate the composition of the gut mycobiota^38–41^ and several diet-related factors have been shown to affect the composition of the gut microbiota of *Milieu Intérieur* subjects.^42^ The association between the snacking habits of the subjects and higher intestinal carriage of *C. albicans* can perhaps be explained by the fact that snacking is often associated with the consumption of overall “unhealthy” food with high sugar and fat contents, and that these factors have been frequently associated with higher *C. albicans* carriage.^38^ The association between a low-sodium diet and *C. albicans* carriage might be more surprising. However, high salt intake has been previously linked to an increase in Th17/IL-17 immune responses that can affect the composition of the gut microbiota.^43^

Finally, we showed that harboring higher levels of *C. albicans* in the gut might be advantageous for the host, as the extent of intestinal *C. albicans* carriage is significantly associated with the expression of *NLRP3* and the levels of IL-2 and CXCL5 after *C. albicans* whole blood stimulation. The NLRP3 inflammasome plays a crucial role in the clearance of *C. albicans*,^44^ while CXCL5 drives neutrophil recruitment during the Th17 immune response.^45,46^ However, IL-2, which is mainly secreted by memory T cells, activates Tregs and therefore helps to maintain the equilibrium between inflammatory and anti-inflammatory responses.^47,48^

These results are consistent with previous reports stating that a previous challenge with fungal components, such as β-glucans, results in an increased survival of mice upon a subsequent fungal infection^49^ and that *C. albicans* intestinal colonization confers protection against systemic infections in mice.^50^ In a similar context, recent studies have shown that mucosa-associated fungi might play a protective role in the host by reinforcing intestinal epithelial function, thus preventing infection^51^. Considering that cell wall peptidoglycans from the gut bacterial microbiota can cross the intestinal epithelial barrier and have been shown to disseminate in the blood of mice (Wheeler *et al*. 2023), it is possible that a similar phenomenon occurs with *C. albicans* cell wall molecules. Subjects highly colonized with *C. albicans* would therefore be more likely to have fungal particles crossing the gut barrier, thus enhancing host immunity against *C. albicans.*

Taken together, these results offer a better understanding of the factors that might affect *C. albicans* colonization in healthy hosts. To the best of our knowledge, this study is the first to establish a potential genetic component of host susceptibility to *C. albicans* intestinal colonization in healthy individuals, which could improve our understanding of this colonization. Moreover, we showed that higher intestinal carriage of *C. albicans* induces a stronger immune response when the host is challenged with this yeast, which suggests a potential protective role of higher *C. albicans* carriage in the gut.

Although the relative importance of some of the associations that we identified here remains to be further explored, these findings pave the way for new intervention strategies to curb the intestinal proliferation of *C. albicans* and thus prevent the emergence of life-threatening infections in high-risk patients.

## Materials & Methods

### The Milieu Intérieur cohort

One thousand healthy volunteers (500 men and 500 women) were recruited from the Rennes area (Ille-et-Vilaine, Bretagne, France) by BioTrial (Rennes, France) between September 2012 and August 2013. The age of the participants was evenly distributed between 20 and 69 years, with 200 people in each decade of life. To minimize the influence of the population substructure, the study was restricted to individuals of self-reported metropolitan French origin for three generations (i.e., with parents and grandparents born in continental France). The participants were selected based on strict inclusion and exclusion criteria.^12^ In short, the subjects had no evidence of severe, chronic, and/or recurrent pathology, and their BMI was limited to ≥ 18.5 and ≤32 kg/m^2^. In addition, subjects were excluded from the study if they (i) had been treated with antibiotics within the last 3 months prior to inclusion; (ii) were HIV or hepatitis C seropositive; (iii) reported having traveled to tropical or subtropical countries in the 6 months prior to inclusion; (iv) were vaccinated shortly before inclusion; (v) were alcohol abusers; (vi) were on a diet prescribed by a doctor or a dietician for medical reasons; or (vii) had food intolerance or allergy. Moreover, only pre- or postmenopausal women were included to avoid the influence of hormonal fluctuations during the premenopausal phase.

The clinical study was approved by the Comité de Protection des Personnes – Ouest 6 on June 13, 2012, and by the French Agence Nationale de Securité du Médicament on June 22, 2012, and was performed in accordance with the Declaration of Helsinki. The study was sponsored by the Institut Pasteur (Pasteur ID-RCB no. 2012-A00238-35) and was conducted as a single-center study without any investigational product. The original protocol was registered at ClinicalTrials.gov (study number: NCT01699893). Informed consent was obtained from the participants after the nature and possible consequences of the study had been explained. The samples and data used in this study were formally established as the Milieu Interieur biocollection (NCT03905993), with approval from the Comité de Protection des Personnes – Sud Mediterranée and the Commission Nationale de l’Informatique et des Libertés on April 11, 2018.

### Fecal DNA extraction

Eight hundred and twenty one fecal samples from *Milieu Intérieur* were available for this study and were thus extracted using the following method. For each sample, 100–250 mg of stool was processed following the repeated bead beating plus column method described by Yu and Morrison 2004^52^ except that a Bullet Blender (NextAdvance, Troy, NY, USA) was used instead of a Mini-Beadbeater^TM^. The DNA samples were eluted in 100 μL of double-distilled H_2_O. Total fecal DNA levels were measured by Qubit (Invitrogen, USA) using the dsDNA Broad Range Kit (Invitrogen, USA). Only DNA extracts with a total DNA concentration above 50 ng/μL were retained for analysis, leading to a total of 695 samples that were further analyzed.

### Quantitative PCR for detection of total fungal load in DNA from human fecal samples

Fungal DNA was quantified by TaqMan qPCR, as described by Liu *et al*. 2012^53^ using a double dye MGB 5’ 6-FAM-labeled probe (Eurogentec, Belgium). All reactions were performed on a CFX96 Real-Time PCR system (BioRad, USA) under the following conditions: 2 min at 50°C, 10 min at 95°C, 15 s at 95°C, and 1 min at 60°C. The last two steps were repeated for 45 cycles. All the samples were tested in two independent rounds, each time in duplicate. In each plate, a qPCR standard consisting of fungal DNA extract with a concentration gradient spanning from 10^−7^ to 10^−2^ ng/g of DNA was used. The presence of qPCR inhibitors was determined using a duplex internal control *C. albicans* qPCR assay as described below. The fungal load was estimated by dividing the fungal DNA concentration (measured by qPCR) by the total DNA concentration of the sample (measured by the Qubit dsDNA Broad Range protocol), as in Zuo *et al*. 2018.^54^

### *Duplex quantitative PCR for detection of* C. albicans *DNA in DNA from human fecal samples and internal amplification control*

Ten microliters of a 1:10 dilution of the extracted total fecal DNA was used as a template for TaqMan qPCR analysis, using *C. albicans* probe and primers described by Guiver *et al*. 2001^55^ in combination with the Cy®5-QXL®670 Probe system of the Universal Exogenous qPCR Positive Control for TaqMan® Assay (Eurogentec, Belgium), in order to identify samples with qPCR inhibitors.

*C. albicans* primers and probes were used at 100 nM and 400 nM concentrations, respectively. All reactions were performed on a CFX96 Real-Time PCR system (BioRad, USA) under the following conditions: 2 min at 50°C, 10 min at 95°C, 15 s at 95°C, and 1 min at 60°C. The last two steps were repeated for 45 cycles. All the samples were quantified in two independent rounds, each time in duplicate. Samples with qPCR inhibitors at a 1:10 dilution were diluted 1:100 and subjected to a new round of qPCR. In each plate, a qPCR standard consisting of *C. albicans* DNA extract (SC5314) with a concentration gradient spanning from 10^−8^ to 10^−3^ ng/g of DNA was used. A detection threshold of 10^−8^ ng/μL of DNA was used for this assay.

### ITS2-targeted sequencing

Library construction, quality control, and sequencing were performed at Novogene (Beijing, China). PCR amplification of ITS2 regions was performed using ITS3/ITS4 primers,^56^ using Novogene’s pipeline. The PCR products were subjected to 2% agarose gel electrophoresis. PCR products from each sample were pooled, end-repaired, A-tailed, and ligated using Illumina adapters. The library was quantified using Qubit and real-time PCR, and size distribution was estimated using a bioanalyzer. Quantified libraries were pooled and sequenced on an Illumina NovaSeq platform (Novogene, Beijing, China) according to the effective library concentration and data amount required. Paired-end reads were assigned to the samples based on their unique barcodes and truncated by cutting off the barcode and primer sequences. Paired-end reads were merged using FLASH^57^ based on the overlap of the reads.

### ITS2-targeted sequence analysis

ITS2 sequences were analyzed with QIIME 2^TM^ (Quantitative Insights into Microbial Ecology).^58^ A total of 72.2 million sequences were generated from 617 samples, with a mean of 117,193 sequences per sample. The sequences were trimmed based on the quality score using the -p-max-ee and -p-trunc-q parameters of DADA2 set at 2.^59^ Using the DADA2 tool,^59^ the sequences were denoised and dereplicated into ASVs and chimeras were removed. Four samples with less than 3,000 sequences were removed from the analyses. 9539 ASVs were recovered, and we generated a feature table for all the remaining samples. Taxonomic annotation was performed on the feature table using UNITE database (v. 9.0). ASVs that were not annotated as fungi were filtered out, resulting in a total of 2488 ASVs. ASVs that could not be annotated at the species level were submitted to a second round of annotation against the UNITE database, and to a classic BLASTN using the BLAST rRNA/ITS databases. Only hits matched with a similarity above 97% to reference genome were retained. The count tables were normalized using the weighted non-null method used in SHAMAN.^60^

### Shotgun metagenomic sequencing and analysis

Shotgun metagenomic sequences were obtained as described in Byrd *et al*. 2020.^13^

*Microbial gene count table*. Gene count tables were generated using the METEOR software suite^61^ which relies on Bowtie2 for read mapping. First, reads were filtered for low-quality using AlienTrimmer^62^ and reads that aligned to the human genome GRCh38-p13 release (identity >95%) were also discarded. The remaining reads were trimmed to 80 bases and mapped to the Integrated Gut Catalogue 2 comprising 10.4 million genes (IGC2),^63^ and the 8.4 million oral microbial gene catalog.^64^ Unique mapped reads (reads mapped to a unique gene in the catalog) were attributed to their corresponding genes. The shared reads (reads that mapped with the same alignment score to multiple genes in the catalog) were attributed to the ratio of their unique mapping counts of the captured genes. The resulting count table was further processed using R package MetaOMineR v1.31.^65^ To decrease technical bias due to different sequencing depths and avoid any artifact of sample size on low-abundance genes, read counts were ‘rarefied’ using 20 M high-quality reads using a random sampling procedure without replacement. The downsized matrix was finally normalized according to gene length and transformed into a frequency matrix (FPKM normalization).

*Metagenomic Species (MGS) abundance profiles*. IGC2 and oral catalogs were organized into 1990 and 853 Metagenomic Species (MGS, cluster of co-abundant genes), respectively, using MSPminer.^64,66^ After removing duplicated MGS (i.e., MGS present in both catalogs), we were left with 2741 MGS. The relative abundance of an MGS was computed as the mean abundance of its 100 ‘marker’ genes (i.e., the genes that correlated the most altogether). If less than 10% of ‘marker’ genes were seen in a sample, the abundance of the MGS was set to 0.

### Culturing of anaerobic gut bacteria

The anaerobic bacterial species tested included isolates from the Rowett Institute (Aberdeen, UK) and isolates purchased from DSMZ (Braunschweig, Germany) (Supplementary Table S3). The strains were revived anaerobically in Hungate tubes containing M2GSC medium supplemented with 1% “liquid gold” and incubated overnight at 37°C in a static 5% CO_2_ incubator (NuAire, Plymouth, MN, USA). “Liquid gold” is the name given to fermenter run off which is collected after the addition of fecal slurry to a complex medium with the aim to simulating the human colon.^67,68^ A spectrophotometer (Novaspec II, Amersham BioSciences UK Ltd., Little Chalfont, UK) was used to monitor cell growth by measuring the optical density of the cultures at 650 nm (OD_650_).

### Quantification of short-chain fatty acids and lactate in gut bacterial culture supernatants by capillary gas chromatography

The samples were analyzed as described by Ricci *et al*. 2022. Briefly, 1 mL of bacterial culture supernatant was filtered, sterilized, and added to a Sorvall screw-capped tube. 2-ethylbutyric acid (50 μL, 01 M) was added as the internal standard. A two-step extraction of organic acids was performed with HCl (0.5 mL) of HCl and diethyl ether (2 mL). Tertiary butyldimethylsilyl was quantified using capillary gas chromatography (Agilent 6890; Agilent Technologies, Santa Clara). To control the quality of the organic acid extraction, an external standard composed of known concentrations of acetic acid, propionic acid, isobutyric acid, n-butyric acid, iso-valeric acid, n-valeric acid, sodium formate, lithium lactate, and sodium succinate was analyzed with the samples in each gas chromatography run.

### *Assessment of* C. albicans *growth inhibition by gut bacterial supernatants*

*C. albicans* strain SC5314 was grown overnight in NGY medium. *C. albicans* cells were diluted 1:100 in fresh NGY medium. In parallel, gut bacteria of interest were grown anaerobically overnight at 37°C in Hungate tubes with M2GSC medium supplemented with 1% of “liquid gold”. The bacterial cultures were centrifuged to collect supernatants. To remove any residual bacteria, each supernatant was filter-sterilized by transfer through a 0.2 μm syringe-driven filter unit (Millex, Merck Millipore Ltd, Kenilworth, NJ, USA).

One hundred microliters of 1:100 diluted *C. albicans* culture was transferred to 96-well microtiter plates (CoStar, Washington, WA, USA) and incubated anaerobically for 24 h in a temperature-controlled plate reader at 37°C (Epoch 2 Microplate Spectrophotometer, BioTek, Swindon, UK) with 100 μL of the bacterial culture supernatant. Fresh NGY medium, M2GSC medium supplemented with 1% of liquid gold, and PBS were used as controls to assess *C. albicans* growth. For each condition and each technical replicate, *C. albicans* growth was estimated by subtracting the optical density at 600 nm obtained at time 0 from the one measured after 24 h. *C. albicans* growth in fresh M2GSC +1% liquid gold was used as reference, thus corresponding to a 100% survival of the yeast. The experiment was performed with three biological replicates, each with six technical replicates.

### *Assessment of the effects of the gut bacterial supernatants on* C. albicans *morphology*

*C. albicans* cells were collected after the 24 h of incubation with filter-sterilized bacterial supernatant and deposited onto microscope slides. To highlight potential bacterial contamination, each plate was Gram stained. Plates were observed under a Leica CME light microscope (Leica Microsystems, Germany) at 100× and 1000× magnification. For each biological replicate, the number of hyphae in two technical replicates was assessed visually in five different sections of the microscopy plates, at 1000× magnification.

### Dietary, medical, environmental, and demographic data

Multiple dietary, medical, environmental, and demographic data were collected for each *Milieu Intérieur* subject in an electronic case report form.^12^ Subjects reported their family medical history, personal medical history, birthplace, smoking, sleeping habits, etc., and completed a food-frequency questionnaire.^42^ In addition, clinical chemistry and hematologic and serologic assessments were performed on the blood of the subjects at the certified Laboratoire de Biologie Médicale, Centre Eugene Marquis (Rennes, France).^13^ After manual curation of the variables that displayed near-zero variance, 201 variables were analyzed for their association with *C. albicans* intestinal colonization.

### Genotyping, quality control, and imputation

The 1000 subjects of the *Milieu Intérieur* cohort were genotyped using the HumanOmniExpress-24 BeadChip (Illumina, California, USA), as described by Patin *et al*. 2018.^19^ SNPs that (i) were unmapped on dgSNP138, (ii) were duplicated, (iii) had a low genotype clustering quality (GenTrain score < 0.35), (iv) had a call rate inferior than 99%, (v) that were monomorphic, (vi) were on a sex chromosome, and/or (vii) were in Hardy-Weinberg disequilibrium (p-value <10^−7^) were removed from the analyses. Possible pairs of genetically related subjects were detected as described by Patin *et al*. 2018.^19^ Genotype imputation was performed as previously described by Patin *et al*. 2018.^19^

### Genome-wide association analysis

We conducted GWAS analyses for *C. albicans* intestinal colonization state (576 colonized and 119 non-colonized) using the linear mixed model implemented in GEMMA,^69^ a mixed model that allows the control of genetic relatedness among donors. For each chromosome, a genetic relatedness matrix (GRM) was estimated based on the remaining 21 chromosomes (‘leave-one-chromosome’ approach, Yang *et al*. 2014). Both genome-wide association analyses were conducted using age, sex, and smoking habits of the subjects as covariates.^70^

Summary statistics were downloaded from the NHGRI-EBI GWAS Catalog^29^ on 19/01/2023 for study GCST90002322 and GCST90002326.^30^

### Whole-Blood TruCulture Stimulation - gene expression and proteomics analysis

TruCulture tubes were prepared in two batches (A and B) with heat-killed *C. albicans* cells (Invitrogen, San Diego, USA) in 2 mL buffered media. *C. albicans* blood stimulation was performed with 1 mL of whole blood for 22 h as previously described.^24,71^ Gene expression analysis was conducted as previously described.^24,72^ The levels of cytokines present in whole blood were measured using Luminex *x*MAP technology, as described previously.^71^

### Biostatistical analyses

All analyses were performed using the R^73^ software (v. 4.1.2). We used the vegan package^74^ (v. 2.6–4) to compute diversity indexes, the Maaslin2 package^14^ (v. 1.6–0) to identify the association between *C. albicans* carriage and microbiota composition, the caret package^75^ (v. 6.0–93) to compute the near-zero variances of the studied variables, and the ggplot2 package^76^ (v. 3.4.0) to generate the figures.

#### *Identification of bacterial species with potential anti-*C. albicans *activity*

Null values of *C. albicans* DNA levels were replaced by the minimal non-null value of the given variable divided by two to allow log10 transformation. MGS with a near-zero variance^75,77^ were filtered out from the analyses. MaAsLin2^14^ was used to search for associations between *C. albicans* levels, deduced from qPCR quantification, and bacterial species relative abundance, deduced from shotgun metagenomics data and annotated at the species level. The age and sex of the subjects were set as random effects in the analysis. Thresholds of prevalence of 1% and of relative abundance of 0.01% were used in MaAsLin2, and the data were log10 transformed. We used a type I error p-value of 0.05 and corrected the p-values for multi-testing using false discovery rate correction (Benjamini-Hochberg).

#### *Identification of fungal species with potential anti-*C. albicans *activity*

The null values of *C. albicans* DNA levels were replaced by the minimal non-null value of the given variable divided by two to allow log10 transformation. Fungal species with near-zero variance^75,77^ were excluded from the analyses. MaAsLin2^14^ was used to search for associations between *C. albicans* colonization state, deduced from qPCR quantification, and fungal species abundance, deduced from ITS2 sequencing data and annotated at the species level. Age, sex of subjects, and technical sequencing variables were set as random effects in the analysis. Thresholds of prevalence of 1% and of relative abundance of 0.01% were used in MaAsLin2, and the data were log10 transformed. We used a type I p-value error of 0.05 and corrected the p-values for multiple testing using false discovery rate correction (Benjamini-Hochberg). In contrast to the analysis of the bacterial species described above, we looked for associations between the relative abundance of the fungal species and *C. albicans* colonization state, rather than the extent of this colonization. This was done to reduce noise in the analysis due to the high variability of the gut mycobiota.

#### *Identification of diet, medical and environmental variables associated with* C. albicans *intestinal carriage*

After manual curation of the variables that displayed near-zero variance, 201 variables were analyzed individually for their association with *C. albicans* intestinal colonization. We used linear mixed models and multiway ANOVA, adjusted for age, sex, and date of collection of fecal samples, to predict either the state of *C. albicans* gut colonization of the subjects or the extent of *C. albicans* intestinal carriage. The null values of *C. albicans* DNA levels were replaced by the minimal non-null value of the given variable divided by two to allow log10 transformation. We used a type I p-value error of .05 and corrected the p-values for multiple testing using false discovery rate correction (Benjamini-Hochberg).

#### *Identification of associations between the extent of* C. albicans *intestinal carriage and the subject’s immune response*

To predict the expression of each gene and the concentration of each cytokine based on *C. albicans* carriage, we used linear mixed models adjusted for the subject’s age, sex, immune cell proportion, genotype at the rs12567990 SNP, and the batch of TruCulture tube used before and after *C. albicans* blood stimulation. We analyzed 13 cytokines and 546 genes. The 546 genes analyzed were previously shown to be differentially expressed upon *C. albicans* blood stimulation.^24^ We used a type I p-value error of 0.05 and corrected the p-values for multiple testing using false discovery rate correction (Benjamini-Hochberg). The contribution of each variable to the expression of *NLRP3* and the levels of CXCL5 and IL-2 was calculated by averaging over the sums of squares in all orderings of the variables in the linear model using the lmg metric in the relaimpo R package.^78^ Averaging over orderings was performed to avoid bias due to correlations among predictors.

## Supplementary Material

Supplemental Material

## Data Availability

The data supporting the conclusions of this article are available in the European Genome-Phenome Archive under the accession codes EGAS00001002460 (https://www.ebi.ac.uk/ega/studies/EGAS00001002460) for the human genotype data and EGAS00001004437 (https://ega-archive.org/studies/EGAS00001004437) for the shotgun metagenomic sequences. ITS2-sequencing data and cytokine concentrations are available on Owey at the following DOI (10.48802/owey.0tnx-g641 for the ITS2 data and doi/10.48802/owey.g54a-kk20 for the cytokine concentrations data). The fungal load and *C. albicans* DNA level data are available in Supplementary Table S4. The associated demographic, lifestyle, environmental, and biochemical metadata can be obtained by contacting the coordinators of the consortium.

## Milieu Intérieur Consortium

†The Milieu Intérieur Consortium^¶^ is composed of the following team leaders: Laurent Abel (Hôpital Necker), Andres Alcover, Hugues Aschard, Philippe Bousso, Nollaig Bourke (Trinity College Dublin), Petter Brodin (Karolinska Institutet), Pierre Bruhns, Nadine Cerf-Bensussan (INSERM UMR 1163 – Institut Imagine), Ana Cumano, Christophe D’Enfert, Ludovic Deriano, Marie-Agnès Dillies, James Di Santo, Gérard Eberl, Jost Enninga, Jacques Fellay (EPFL, Lausanne), Ivo Gomperts-Boneca, Milena Hasan, Gunilla Karlsson Hedestam (Karolinska Institutet), Serge Hercberg (Université Paris 13), Molly A Ingersoll (Institut Cochin and Institut Pasteur), Olivier Lantz (Institut Curie), Rose Anne Kenny (Trinity College Dublin), Mickaël Ménager (INSERM UMR 1163 – Institut Imagine), Frédérique Michel, Hugo Mouquet, Cliona O’Farrelly (Trinity College Dublin), Etienne Patin, Antonio Rausell (INSERM UMR 1163 – Institut Imagine), Frédéric Rieux-Laucat (INSERM UMR 1163 – Institut Imagine), Lars Rogge, Magnus Fontes (Institut Roche), Anavaj Sakuntabhai, Olivier Schwartz, Benno Schwikowski, Spencer Shorte, Frédéric Tangy, Antoine Toubert (Hôpital Saint-Louis), Mathilde Touvier (Université Paris 13), Marie-Noëlle Ungeheuer, Christophe Zimmer, Matthew L. Albert (Hifibio), Darragh Duffy^§^, Lluis Quintana-Murci^§^,

¶ Unless otherwise indicated, partners are located at the Institut Pasteur, Paris.

^§^ Co-coordinators of the Milieu Intérieur Consortium.

Additional information can be found at: http://www.milieuinterieur.fr.
