# Supplementary material for: Unveiling Candida albicans intestinal carriage in healthy volunteers: the role of micro- and mycobiota, diet, host genetics and immune response
Source: Gut Microbes. 2023 Nov 28;15(2):2287618. doi: 10.1080/19490976.2023.2287618 (PMC10732203; doi:10.1080/19490976.2023.2287618)
Supplement: Supplemental Material [file KGMI_A_2287618_SM2805.zip › SupplementaryFigure1.docx]

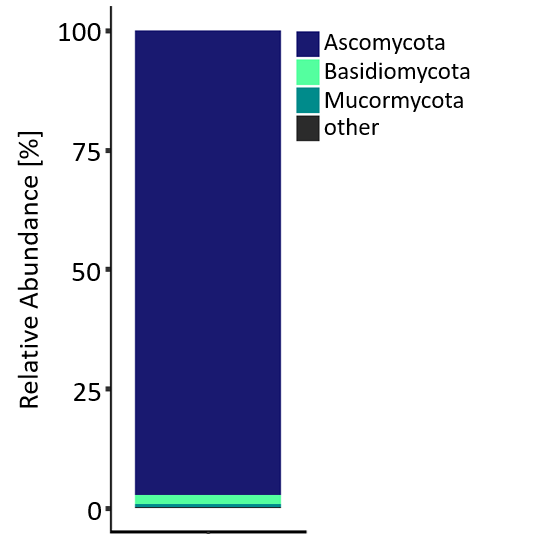


Supplementary Figure 1: Phyla composition of healthy subjects. *Barplot of the average relative abundance of the main fungal phyla for 604 healthy subjects. The fungal phyla represented have a mean relative abundance above 0.01%*
