# Supplementary material for: Unveiling Candida albicans intestinal carriage in healthy volunteers: the role of micro- and mycobiota, diet, host genetics and immune response
Source: Gut Microbes. 2023 Nov 28;15(2):2287618. doi: 10.1080/19490976.2023.2287618 (PMC10732203; doi:10.1080/19490976.2023.2287618)
Supplement: Supplemental Material [file KGMI_A_2287618_SM2805.zip › SupplementaryFigure2.docx]

**
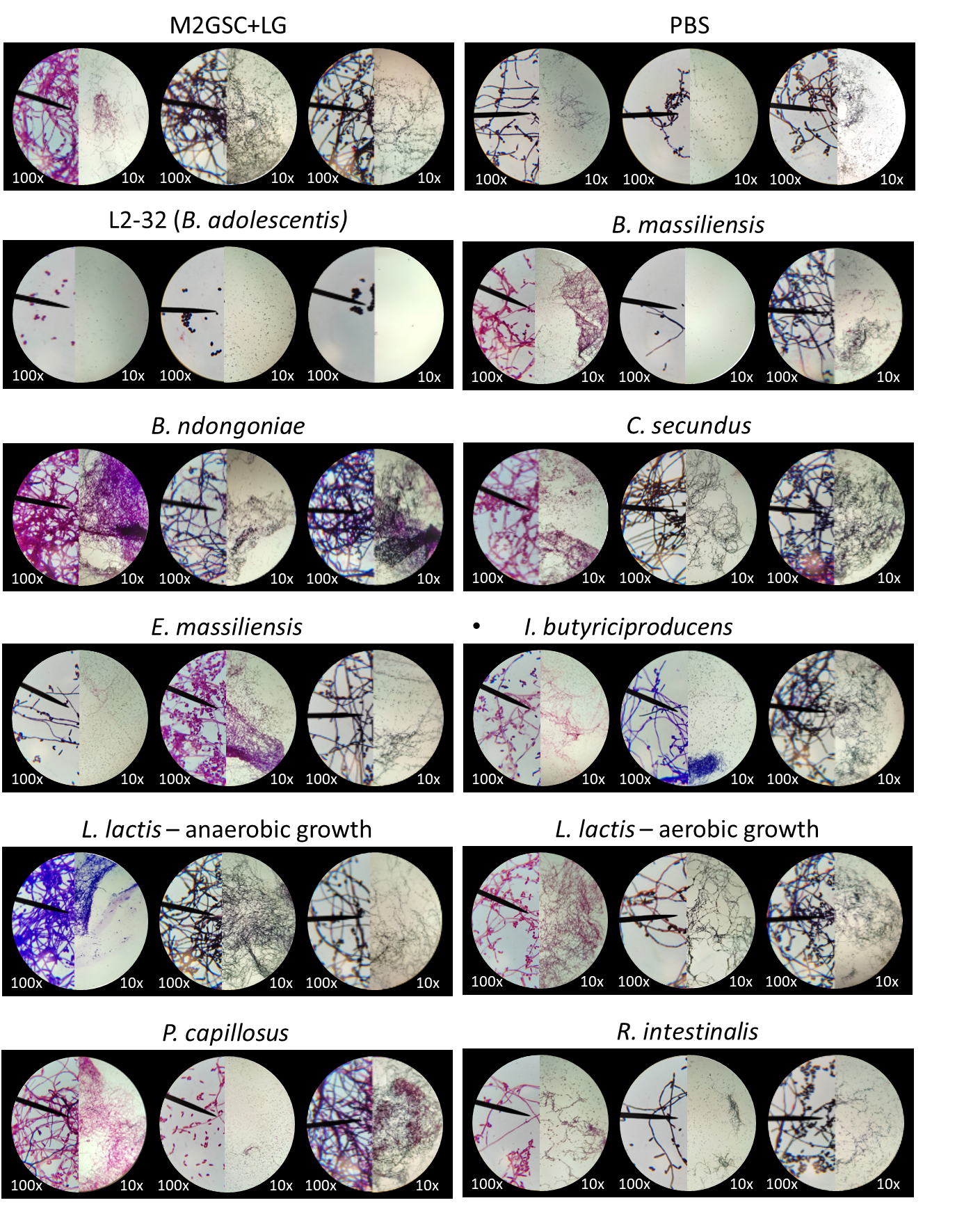
**

Supplementary Figure 2: Gram staining and light microscopy of *C. albicans* SC5314 cells after 24 h of exposure to the supernatants from gut bacterial isolates. *The bacterial growth medium, M2GSC+LG, was used as media control, phosphate buffered saline (PBS) solution was used as no-media control and supernatant of* Bifidobacterium adolescentis *strain L2-32, was used as a filamentation inhibition positive control.*
