# Supplementary material for: Unveiling Candida albicans intestinal carriage in healthy volunteers: the role of micro- and mycobiota, diet, host genetics and immune response
Source: Gut Microbes. 2023 Nov 28;15(2):2287618. doi: 10.1080/19490976.2023.2287618 (PMC10732203; doi:10.1080/19490976.2023.2287618)
Supplement: Supplemental Material [file KGMI_A_2287618_SM2805.zip › SupplementaryTable1.docx]

Supplementary Table 1. Prevalence and mean relative abundance of the fungal species that were detected in at least 50% of the 604 studied healthy subjects with a relative abundance above 0.1%. *The prevalence and relative abundance were obtained by ITS2-targeted sequencing.*

|  | **Prevalence [%]** | **Mean relative abundance [%] (min, max)** |
| --- | --- | --- |
| *Saccharomyces cerevisiae* | 97.7 | 45.3 (0, 99.3) |
| *Geotrichum candidum* | 97.5 | 21.4 (0, 99.8) |
| *Penicillium roqueforti* | 74.2 | 7.3 (0, 98.5) |
| *Debaryomyces hansenii* | 66.9 | 2.3 (0, 85.1) |
| *Candida albicans* | 56.3 | 1.7 (0, 88.0) |
