# Supplementary material for: Unveiling Candida albicans intestinal carriage in healthy volunteers: the role of micro- and mycobiota, diet, host genetics and immune response
Source: Gut Microbes. 2023 Nov 28;15(2):2287618. doi: 10.1080/19490976.2023.2287618 (PMC10732203; doi:10.1080/19490976.2023.2287618)
Supplement: Supplemental Material [file KGMI_A_2287618_SM2805.zip › SupplementaryTable2.docx]

Supplementary Table 2: List of the diet, medical and lifestyle variables analyzed for an association with *C. albicans* carriage and colonization.

| **VARIABLE NAME** | **PANEL** | **DESCRIPTION** | **TYPE** | **VALUE** |
| --- | --- | --- | --- | --- |
| *ABDOCM.V0* | Basic physiological measurements | Abdominal circumference Visit 1 | double | cm |
| *ACCOUCH* | Personal medical history at birth | Delivery time | binary | 0=Born at term; 1=Premature |
| *AGE* | Demographics | Age in years | integer | years |
| *ALB.V0* | Laboratory measure | Biochemistry: Albumin | double | g/L |
| *ALCF* | Food and nutrition | Glasses of spirit per week | integer | glasses/week |
| *ALCOOL* | Food and nutrition | Alcohol | integer | 30, 12, 4, 2, 0, per day |
| *ALIM* | Personal medical history at birth | Mode of feeding at birth | binary | 0=Maternal breastfeeding; 1=Formula milk |
| *ALLER* | Family medical history | Allergic disease | binary | 0=No; 1=Yes |
| *ALP.V0* | Laboratory measure | Biochemistry: Alkaline phosphatases | double | IU/L |
| *ALT.V0* | Laboratory measure | Biochemistry: ALAT | double | IU/L |
| *AMIDI* | Food and nutrition | Eat only in the afternoon | binary | 0 = no, 1 = yes |
| *APPET* | Sleep habits, drug habits, and psychological problems | Little or too much appetite, last 2 weeks | integer | 0, 3, 8, 14 days the last two weeks |
| *APPLOC* | Food and nutrition | Essential oils by local application | integer | 0, 1, 5, 10, 30 times per month |
| *AST.V0* | Laboratory measure | Biochemistry: ASAT | double | IU/L |
| *ATTAQUE* | Family medical history | Cerebral accident, hemorrhage or congestion | binary | 0=No; 1=Yes |
| *AUTCFIL* | Family medical history | Relationship to Other cancer | categorical | 0=no relationship; 1=Father; 2=Mother; 3=Brother; 4=Sister; 5=Father & Mother; 6=Father & Brother; 9=Mother & Sister; NA=missing data |
| *BASO.V0* | Laboratory measure | Hematology: Basophils | double | G/L |
| *BICARB.V0* | Laboratory measure | Biochemistry: Bicarbonates | double | mmol/L |
| *BIERE* | Food and nutrition | Glasses of beer/cider per week | integer | glasses/week |
| *BILI.V0* | Laboratory measure | Biochemistry: Total bilirubin | double | µmol/L |
| *BLANC* | Biometrics | Hair graying | binary | 0=no; 1=yes |
| *BLANCA* | Biometrics | Age at which hair started graying | integer | years |
| *BMI.V0* | Basic physiological measurements | BMI (kg/m^2) Visit 1 | double | kg/m^2 |
| *BRUIT* | Socio-professional information | Exposure to noise | categorical | 0=No exposure; 1=Past exposure; 2=Current exposure |
| *CAUTRE* | Family medical history | Other cancer | binary | 0=No; 1=Yes |
| *CA.V0* | Laboratory measure | Biochemistry: Calcium | double | mmol/L |
| *CCOLON* | Family medical history | Colon/rectum cancer | binary | 0=No; 1=Yes |
| *CHARCU* | Food and nutrition | Cooked and cured meats (ham, salami, pate etc.) | integer | 0, 1, 5, 10, 30, 60 times per month |
| *CHEVEUX* | Biometrics | Hair color | integer | 1=Black; 2=Dark brown; 3=Light brown; 4=Red; 5=Blond; 6=Other |
| *CHOL.V0* | Laboratory measure | Biochemistry: Total cholesterol | double | mmol/L |
| *CL.V0* | Laboratory measure | Biochemistry: Chloride | double | mmol/L |
| *CMSEQ* | Concomitant drug treatment | Subject takes concomitant drug treatment(s) | integer | Number of different drug treatments |
| *CMV.V0* | Laboratory measure | Serology: CMV | binary | 0=negative; 1=positive |
| *CONCEN* | Sleep habits, drug habits, and psychological problems | Difficulty concentrating on things like reading the newspaper or watching the television, last 2 weeks | integer | 0, 3, 8, 14 days the last two weeks |
| *CORREC* | Biometrics | Corrective lenses worn | binary | 0=no; 1=yes |
| *CORRECP* | Biometrics | Type of corrective lenses | binary | 1=glasses; 2=contact lenses |
| *CREAT.V0* | Laboratory measure | Biochemistry: Creatinine | double | µmol/L |
| *CRP.V0* | Laboratory measure | Biochemistry: CRP | double | mg/L |
| *CRUD* | Food and nutrition | Raw vegetables | integer | 0, 1, 5, 10, 30, 60 times per month |
| *CSEIN* | Family medical history | Breast cancer | binary | 0=No; 1=Yes |
| *CSP* | Socio-professional information | Socio-professional category, if ever employed | categorical | 1=Farmer; 2=Artisan, tradesman, company director; 3=Senior executive, independent profession; 4=Middle management; 5=Employee; 6=Labourer; 7=Other (e.g. artist, clergy, soldier, policeman); NA=missing data; NaN=not applicable |
| *CUISIN* | Food and nutrition | Cooking | integer | 1, 5, 20, times per month |
| *DEJ* | Food and nutrition | Eats lunch | integer | 0 = always, 1 = not always, 2 = never |
| *DESS* | Food and nutrition | Desserts (cream desserts, ice cream, cream cakes, etc.) | integer | 0, 1, 5, 10, 30, 60 times per month |
| *DIABETE* | Family medical history | Diabetes | binary | 0=No; 1=Yes |
| *DIABP1.V0* | Basic physiological measurements | Diastolic measure 1 (mmHg) Visit 1 | double | mmHg |
| *DIABP2.V0* | Basic physiological measurements | Diastolic measure 2 (mmHg) Visit 1 | double | mmHg |
| *DIAFIL* | Family medical history | Relationship to Diabetes | categorical | 0=no relationship; 1=Father; 2=Mother; 3=Brother; 4=Sister; 5=Father & Mother; 6=Father & Brother; 8=Mother & Brother; NA=missing data |
| *DIFF* | Sleep habits, drug habits, and psychological problems | Difficulties falling asleep or staying asleep, or sleeping too much, last 2 weeks | integer | 0, 3, 8, 14 days the last two weeks |
| *DIFFATM* | Food and nutrition | Essential oils by diffusion into the atmosphere | integer | 0, 1, 5, 10, 30 times per month |
| *DINER* | Food and nutrition | Eats dinner | integer | 0 = always, 1 = not always, 2 = never |
| *DORDIF* | Sleep habits, drug habits, and psychological problems | Does the subject often find it difficult to fall asleep or to remain asleep? | integer | 0,1,2,3 = Never, Sometimes, occasionally, most of the time, all the time |
| *DORH.T1* | Sleep habits, drug habits, and psychological problems | Hours of sleep, in decimal | double | hours/day |
| *DORLUM* | Sleep habits, drug habits, and psychological problems | On average, how much light enters the subject’s bedroom while he/she is asleep? | integer | 0,1,2 = Never, Sometimes, occasionally, most of the time, all the time |
| *EMPLOIP* | Socio-professional information | Category of unemployment | categorical | 1=Student; 2=Looking for first job; 3=Unemployed; 4=Housewife/househusband; 5=Retired; NA=missing data; NaN=not applicable |
| *EMPLOIS* | Socio-professional information | Steady job | binary | 0=No; 1=Yes |
| *EOS.V0* | Laboratory measure | Hematology: Eosinophils | double | G/L |
| *FASTF* | Food and nutrition | Fast-food restaurants | integer | 0, 1, 5, 20 times per month |
| *FATI* | Sleep habits, drug habits, and psychological problems | Feeling tired or having little energy, last 2 weeks | integer | 0, 3, 8, 14 days the last two weeks |
| *FCRUS* | Food and nutrition | Raw fruit | integer | 0, 1, 5, 10, 30, 60 times per month |
| *FCUITS* | Food and nutrition | Cooked fruit (stewed fruit etc.) | integer | 0, 1, 5, 10, 30, 60 times per month |
| *FC.V0* | Basic physiological measurements | Heart rate (bpm) Visit 1 | double | bpm |
| *FECUL* | Food and nutrition | Starchy foods (pasta, rice, potatoes etc.) | integer | 0, 1, 5, 10, 30, 60 times per month |
| *FRITS* | Food and nutrition | Fried products (chips, crisps, doughnuts, nuggets, cordon bleu, etc.) or pasties/pies | integer | 0, 1, 5, 10, 30, 60 times per month |
| *FROM* | Food and nutrition | Cheese | integer | 0, 1, 5, 10, 30, 60 times per month |
| *FSH.V0* | Laboratory measure | Biochemistry: Follicle stimulating hormone | double | U/L |
| *GFR.V0* | Laboratory measure | Biochemistry: Glomerular filtration rate | double | mL/min/1.73m2 |
| *GGT.V0* | Laboratory measure | Biochemistry: Gamma GT | double | IU/L |
| *GLUC.V0* | Laboratory measure | Biochemistry: Fasting glycaemia | double | mmol/L |
| *GRIGN* | Food and nutrition | Nibble between meals | categorical | 0 = never, 1 = sometimes, 2 = often |
| *GRIPPE.V1* | Laboratory measure | Serology: Influenza | binary | 0=negative; 1=positive |
| *GROSS* | Visit scheme | Pregnancy urine test carried out | binary | 0=no; 1=yes |
| *HASCHICH* | Sleep habits, drug habits, and psychological problems | Hashish | integer | 0 = never, 1 = rarely, 2 = regularly |
| *HCT.V0* | Laboratory measure | Hematology: Hematocrit | double | RATIO |
| *HDL.V0* | Laboratory measure | Biochemistry: HDL | double | mmol/L |
| *HEIGHT.V0* | Basic physiological measurements | Height (cm) Visit 1 | double | cm |
| *HGB.V0* | Laboratory measure | Hematology: Hemoglobin | double | g/dL |
| *HYPERT* | Family medical history | Arterial hypertension | binary | 0=No; 1=Yes |
| *HYPFIL* | Family medical history | Relationship to Arterial hypertension | categorical | 0=no relationship; 1=Father; 2=Mother; 3=Brother; 4=Sister; 5=Father & Mother; 6=Father & Brother; 7=Father & Sister; 8=Mother & Brother; 9=Mother & Sister; 10=Father & Mother & Sister |
| *IGA.V1* | Laboratory measure | Immunology: IgA | double | g/l |
| *IGE.V1* | Laboratory measure | Immunology: IgE | double | UI/ml |
| *IGG.V1* | Laboratory measure | Immunology: IgG | double | g/l |
| *IGM.V1* | Laboratory measure | Immunology: IgM | double | g/l |
| *INACT* | Socio-professional information | Time without professional activity, if unemployed or retired | categorical | 0=Never worked; 1=0-1 year; 2=1-3 years; 3=3 and more years; NA=missing data |
| *INFARC* | Family medical history | Myocardial infarction | binary | 0=No; 1=Yes |
| *INFFIL* | Family medical history | Relationship to Myocardial infarction | categorical | 0=no relationship; 1=Father; 2=Mother; 3=Brother; 5=Father & Mother; 6=Father & Brother |
| *K.V0* | Laboratory measure | Biochemistry: Potassium | double | mmol/L |
| *LAIT* | Food and nutrition | Dairy products (milk, yoghurt etc.) | integer | 0, 1, 5, 10, 30, 60 times per month |
| *LDL.V0* | Laboratory measure | Biochemistry: LDL | double | mmol/L |
| *LEGC* | Food and nutrition | Cooked vegetables | integer | 0, 1, 5, 10, 30, 60 times per month |
| *LEGS* | Food and nutrition | Dried pulses (lentils, chickpeas, split peas etc.) | integer | 0, 1, 5, 10, 30, 60 times per month |
| *LOG* | Demographics | Does the subject own his/her housing? | binary | 1=landlord; 2=leaser |
| *LYM.V0* | Laboratory measure | Hematology: Lymphocytes | double | G/L |
| *MATIN* | Food and nutrition | Eat only in the morning | binary | 0 = no, 1 = yes |
| *MCHC.V0* | Laboratory measure | Hematology: Mean corpuscular hemoglobin concentration | double | g/L |
| *MCH.V0* | Laboratory measure | Hematology: Mean corpuscular hemoglobin | double | pg |
| *MCV.V0* | Laboratory measure | Hematology: Mean corpuscular volume | double | µm3 |
| *MEDACT.LOCALCON* | Concomitant drug treatment | Subject takes a drug treatment whose action is: contraception (local) | binary | 0=no; 1=yes |
| *MEDACT.ORALCONT* | Concomitant drug treatment | Subject takes a drug treatment whose action is: oral contraception | binary | 0=no; 1=yes |
| *MEDCAT* | Concomitant drug treatment | General category of drug treatment(s) | categorical | 1=female sex hormons; 2=cardiovascular treatment; 3=thyroid hormon replacement; 4=Miscellaneous |
| *MEDIND.CONTRACE* | Concomitant drug treatment | Subject takes a drug treatment whose indication is: Contraception | binary | 0=no; 1=yes |
| *METABOSCORE* | Laboratory measure | Metabolic score, estimated as described in Thomas et al., Clin Immunol 2015 | integer | Number of risk factors for the Metabolic Syndrome, ranging from 0 to 5 |
| *MHCAT10.MHTESTYN2* | Medical history | Cat:10: Tonsillectomy | binary | 0=No; 1=Yes |
| *MHCAT10.MHTESTYN3* | Medical history | Cat:10: Appendicectomy | binary | 0=No; 1=Yes |
| *MHCAT10.MHTESTYN9* | Medical history | Cat:10: Other | binary | 0=No; 1=Yes |
| *MHCAT10.ORTHO* | Medical history | Cat:10: Orthopedic and maxillofacial surgery | binary | 0=No; 1=Yes |
| *MHCAT10.REPRO* | Medical history | Cat:10: Reproductive system surgery | categorical | 0=No; 1=Yes; 2=Yes, significant surgery (ovariectomy, ectopic testis) |
| *MHCAT10.TEETH* | Medical history | Cat:10: Teeth extraction | binary | 0=No; 1=Yes |
| *MHCAT10.VASC* | Medical history | Cat:10: Vascular system surgery | categorical | 0=No; 1=Yes |
| *MHCAT10.VISC* | Medical history | Cat:10: Visceral system surgery | categorical | 0=No; 1=Yes; 2=Yes, significant surgery (cholecystectomy, thyroidectomy) |
| *MHCAT11.MHTESTYN1* | Medical history | Cat:11: Measles | binary | 0=No; 1=Yes |
| *MHCAT11.MHTESTYN3* | Medical history | Cat:11 :Hepatitis B | binary | 0=No; 1=Yes |
| *MHCAT11.MHTESTYN5* | Medical history | Cat:11: Flu | binary | 0=No; 1=Yes |
| *MHCAT8.MHTESTYN1* | Medical history | Cat:8: Measles | binary | 0=No; 1=Yes, antecedent |
| *MHCAT8.MHTESTYN2* | Medical history | Cat:8: Rubella | binary | 0=No; 1=Yes, antecedent |
| *MHCAT8.MHTESTYN3* | Medical history | Cat:8: Chicken pox | categorical | 0=No; 1=Yes, antecedent; 2=Yes, current w/o treatment |
| *MHCAT8.MHTESTYN4* | Medical history | Cat:8: Mumps | binary | 0=No; 1=Yes, antecedent |
| *MHCAT8.MHTESTYN9* | Medical history | Cat:8: Other | binary | 0=No; 1=Yes, antecedent |
| *MHCATYN10* | Medical history | Surgical interventions (Cat:10) | binary | 0=No; 1=Yes |
| *MHCATYN8* | Medical history | Childhood diseases (Cat:8) | binary | 0=No; 1=Yes |
| *MIN* | Food and nutrition | Minerals only | integer | 0, 1, 5, 10, 30 times per month |
| *MONO.V0* | Laboratory measure | Hematology: Monocytes | double | G/L |
| *MULTI* | Food and nutrition | Multimineral and multivitamins | integer | 0, 1, 5, 10, 30 times per month |
| *MUMPS* | Vaccination history | Vaccination against mumps | binary | 0=No; 1=Yes |
| *NAISSP* | Personal medical history at birth | Weight at birth | double | kg |
| *NAISST* | Personal medical history at birth | Length at birth | double | cm |
| *NBYLPSEXP* | Smoking habits | Number of years since last secondhand smoking exposure | integer | Years |
| *NBYLTABAC* | Smoking habits | Number of years since last smoke | integer | Years |
| *NBYPSEXP* | Smoking habits | Number of years exposed to secondhand smoking | integer | Years |
| *NBYTABAC* | Smoking habits | Number of years smoking | integer | Years |
| *NCOLL* | Food and nutrition | Eats no snacks | binary | 0 = no, 1 = yes |
| *NEUT.V0* | Laboratory measure | Hematology: Neutrophils | double | G/L |
| *NIVETUD* | Socio-professional information | Level of education | categorical | 1=No diploma; 2=Primary school certificate; 3=CAP, BEP, Brevet de colleges; 4=Baccalaureat; 5=Higher education, cycle 1 (DUT, BTS, DEUG, L2); 6=Higher education, cycle 2 and 3 (L3, M1, M2, PhD) |
| *NVILLES* | Geographic origin | Number of place(s) of residence (French department) before the age of 13 | integer | places |
| *OEUFS* | Food and nutrition | Eggs | integer | 0, 1, 5, 10, 30, 60 times per month |
| *PAIN* | Food and nutrition | Bread (one baguette ~250 g) | integer | 400, 250, 125, 50, grams of bread per day |
| *PDEJ* | Food and nutrition | Eats breakfast | integer | 0 = always, 1 = not always, 2 = never |
| *PERCEP* | Sleep habits, drug habits, and psychological problems | Poor self-image, or you think that you are a loser or have not achieved your own expectations or those of your family, last 2 weeks | integer | 0, 3, 8, 14 days the last two weeks |
| *PHOS.V0* | Laboratory measure | Biochemistry: Phosphate | double | mmol/L |
| *PHYSDUR* | Demographics | Duration of professional physical activity | categorical | 1=1 to 20 minutes per day; 2=21 to 60 minutes per day; 3=1 to 2 hours per day; 4=More than 2 hours per day |
| *PHYSDUR.T1* | Demographics | Duration of professional physical activity | double | proportion of 8h working day |
| *PHYSJ* | Demographics | Days per week of physical activity during leisure | integer | days per week |
| *PHYSL* | Demographics | Physical activity during leisure | integer | 1=Little or no physical activity; 2=Moderate physical activity; 3=Intense physical activity |
| *PHYSP* | Demographics | Professional physical activity | integer | 1=none; 2=moderate; 3=considerable |
| *PHYST* | Demographics | Hours per day of physical activity during leisure | double | hours per day |
| *PLAIS* | Sleep habits, drug habits, and psychological problems | Lack of interest or pleasure in doing things, last 2 weeks | integer | 0, 3, 8, 14 days the last two weeks |
| *PLATC* | Food and nutrition | Ready meals | integer | 0, 1, 5, 10, 30, 60 times per month |
| *PLAT.V0* | Laboratory measure | Hematology: Platelets | double | G/L |
| *PLVTSEDT.V1.T1* | Visit scheme | Hour at which stool sample was taken | double | hours |
| *PLVTSEYN.V1.T1* | Visit scheme | Date at which stool sample was taken, in days since 09-01-2012 | integer | days |
| *POISSON* | Food and nutrition | Fish | integer | 0, 1, 5, 10, 30, 60 times per month |
| *POUSS* | Socio-professional information | Exposure to dust | categorical | 0=No exposure; 1=Past exposure; 2=Current exposure; NA=Unknown |
| *PRODALL1* | Food and nutrition | Uses reduced sugar products | binary | 0 = no, 1 = yes |
| *PRODALL2* | Food and nutrition | Uses reduced fat products | binary | 0 = no, 1 = yes |
| *PRODALL3* | Food and nutrition | Uses reduced salt products | binary | 0 = no, 1 = yes |
| *PRODALL4* | Food and nutrition | Uses light products | binary | 0 = no, 1 = yes |
| *PROT.V0* | Laboratory measure | Biochemistry: Total proteins | double | g/L |
| *PSEXP* | Smoking habits | Secondhand smoking | categorical | 0 = never been exposed, 1 = exposed in past, 2 = currently exposed |
| *PTOX* | Socio-professional information | Exposure to toxic products | categorical | 0=No exposure; 1=Past exposure; 2=Current exposure; NA=Unknown |
| *RBC.V0* | Laboratory measure | Hematology: Erythrocytes | double | T/L |
| *REPASH* | Food and nutrition | Do you have regular mealtimes during workdays | binary | 0 = have regular mealtimes, 1 = no |
| *RESTAU* | Food and nutrition | Restaurants other than work, or at friends’ houses | integer | 1, 5, 20, times per month |
| *REVENUS* | Socio-professional information | Net monthly income of the household (EUR) | integer | 1=0-1000€; 2=1001-2000€; 3=2001-3000€; 4=3001-4000€; 5=4001-5000€; 6=5001€ and more; NA=missing data |
| *RUBELLA* | Vaccination history | Vaccination against rubella | binary | 0=No; 1=Yes; NA=missing data |
| *SEL* | Food and nutrition | Salt consumption habits | binary | 1 = doesn't salt food, 2 often salts food |
| *SEX* | Demographics | Sex | binary | 1=male; 2=female |
| *SODAS* | Food and nutrition | Sodas and other sugary drinks (other than diet drinks low in sugar) | integer | 0, 1, 5, 10, 30, 60 times per month |
| *SODIUM.V0* | Laboratory measure | Biochemistry: Sodium | double | mmol/L |
| *SOIREE* | Food and nutrition | Eat only during the evening or night | binary | 0 = no, 1 = yes |
| *STRESS* | Sleep habits, drug habits, and psychological problems | Major Negative Life event, loss of loved one etc., last 12 month | binary | 0 = no, 1 = yes |
| *SUCR* | Food and nutrition | Sweet things (chocolate, sweets, honey, jam etc.) | integer | 0, 1, 5, 10, 30, 60 times per month |
| *SYSBP1.V0* | Basic physiological measurements | Systolic measure 1 (mmHg) Visit 1 | double | mmHg |
| *SYSBP2.V0* | Basic physiological measurements | Systolic measure 2 (mmHg) Visit 1 | double | mmHg |
| *TABAC.T1* | Smoking habits | Smoking tobacco? | categorical | Non-smoker = 0, Ex-Smoker = 1, Smoker = 2 |
| *TEMP.V0* | Basic physiological measurements | Ear temperature (°C) Visit 1 | double | °C |
| *TOTNROFCIGS* | Smoking habits | Total number of cigarettes smoked | integer | Cigarettes |
| *TRAVJ* | Socio-professional information | Working time | categorical | 1=Exclusively during the day; 2=Exclusively during the night; 3=Without fixed hours; NA=missing data; NaN=not applicable |
| *TRAVT* | Socio-professional information | Type of employment | binary | 0=Part time; 1=Full time |
| *TRIG.V0* | Laboratory measure | Biochemistry: Triglycerides | double | mmol/L |
| *TRIS* | Sleep habits, drug habits, and psychological problems | Feeling sad, depressed, or despairing, last 2 weeks | integer | 0, 3, 8, 14 days the last two weeks |
| *URATE.V0* | Laboratory measure | Biochemistry: Uric Acid | double | µmol/L |
| *UREA.V0* | Laboratory measure | Biochemistry: Urea | double | mmol/L |
| *VIANDE* | Food and nutrition | Meat | integer | 0, 1, 5, 10, 30, 60 times per month |
| *VIENN* | Food and nutrition | Pastries and sweet breads (croissants, brioches, pains au chocolat, etc.) | integer | 0, 1, 5, 10, 30, 60 times per month |
| *VILLENPOP* | Geographic origin | Number of inhabitants in the place of birth | integer | inhabitants |
| *VILLENPOP20* | Geographic origin | Number of inhabitants in the place of birth larger than 20,000 | binary | 0=no; 1=yes |
| *VILLESPOP* | Geographic origin | Average number of inhabitants in the place(s) of residence before the age of 13 | double | inhabitants |
| *VILLESPOP20* | Geographic origin | Number of inhabitants in at least one place of residence before the age of 13 larger than 20,000 | binary | 0=no; 1=yes |
| *VIN* | Food and nutrition | Glasses of wine per week | integer | glasses/week |
| *VISIT2* | Visit scheme | Presence at follow-up visit | binary | 0=not done; 1=done |
| *VIT* | Demographics | Subject shares housing with... | categorical | 1=Alone with no children; 2=Alone with children; 3=With a partner but no children; 4=With a partner and children |
| *VITA* | Food and nutrition | Vitamins only | integer | 0, 1, 5, 10, 30 times per month |
| *VIT.COUPLE* | Demographics | Subject shares housing with his/her partner | binary | 0=no; 1=yes |
| *VIT.ENFANTS* | Demographics | Subject shares housing with his/her children | binary | 0=no; 1=yes |
| *VOIE* | Personal medical history at birth | Route of delivery | binary | 0=Vaginal delivery; 1=Caesarean section |
| *VORALE* | Food and nutrition | Essential oils by oral route | integer | 0, 1, 5, 10, 30 times per month |
| *WBC.V0* | Laboratory measure | Hematology: Leucocytes | double | G/L |
| *WEIGHT.V0* | Basic physiological measurements | Weight (kg) Visit 1 | double | kg |
| *WHOOPING.COUGH* | Vaccination history | Vaccination against whooping cough | binary | 0=no; 1=yes |
| *YELLOW.FEVER* | Vaccination history | Vaccination against yellow fever | binary | 0=no; 1=yes |
| *YEUX* | Biometrics | Eye color | integer | 1=Gray; 2=Blue; 3=Green/Hazel green; 4=Hazel brown; 5=Light brown; 6=Dark brown; 7=Other |
