# Supplementary material for: Unveiling Candida albicans intestinal carriage in healthy volunteers: the role of micro- and mycobiota, diet, host genetics and immune response
Source: Gut Microbes. 2023 Nov 28;15(2):2287618. doi: 10.1080/19490976.2023.2287618 (PMC10732203; doi:10.1080/19490976.2023.2287618)
Supplement: Supplemental Material [file KGMI_A_2287618_SM2805.zip › SupplementaryTable3.docx]

Supplementary Table 3: List of the gut bacterial strains whose supernatants were tested for inhibitory activity against *C. albicans. Bifidobacterium adolescentis* was used as a positive inhibition control, since previous work has shown this to be a potent inhibitor of *C. albicans*^1^.

| **Strain** | **Species** |
| --- | --- |
| DSM26588 | *Intestinimonas butyriciproducens* |
| DSM17679 | *Bacteroides massiliensis* |
| DSM103636 | *Bacteroides ndongoniae* |
| DSM28864 | *Coprobacter secundus* |
| DSM25476 | *Enorma massiliensis* |
| DSM23940 | *Pseudoflavonifractor capillosus* |
| IL14-03 | *Lactococcus lactis* |
| DSM14610 | *Roseburia intestinalis* |
| L2-32 | *Bifidobacterium adolescentis* |

1. Ricci L, Mackie J, Donachie GE, Chapuis A, Mezerová K, Lenardon MD, Brown AJP, Duncan SH, Walker AW, 2022. Human gut bifidobacteria inhibit the growth of the opportunistic fungal pathogen Candida albicans. *FEMS Microbiol Ecol*; 98.
